# The impact of topical or oral antibiotics in children with acute otitis media on their middle ear, nasopharyngeal, and gut microbiomes

**DOI:** 10.1017/S0950268826101836

**Published:** 2026-06-23

**Authors:** Juana Claus, Ross S. McInnes, Saskia Hullegie, Roger A. M. J. Damoiseaux, Anne G. M. Schilder, Janetta Top, Rob Schuurman, Mei Ling Chu, Debby Bogaert, Willem van Schaik, Roderick P. Venekamp, Janneke H. H. M. van de Wijgert

**Affiliations:** 1 Department of Epidemiology and Health Economics, Julius Center for Health Sciences and Primary Care, University Medical Centre Utrecht, Utrecht University, Utrecht, Netherlands; 2 Institute of Microbiology and Infection and Department of Microbes, Infection and Microbiomes, School of Infection, Inflammation and Immunology, College of Medicine and Health, University of Birmingham, Birmingham, UK; 3 Department of General Practice and Nursing Science, Julius Center for Health Sciences and Primary Care, University Medical Centre Utrecht, Utrecht University, Utrecht, Netherlands; 4 National Institute for Health Research, University College London Hospitals Biomedical Research Centre, London, UK; 5 evidENT, University College London Ear Institute, London, UK; 6 Department of Medical Microbiology, University Medical Centre Utrecht, Utrecht University, Utrecht, Netherlands; 7 Department of Paediatrics, University Medical Centre Utrecht, Utrecht University, Utrecht, Netherlands; 8 Institute for Regeneration and Repair, The University of Edinburgh College of Medicine and Veterinary Medicine, Edinburgh, UK

**Keywords:** acute otitis media, children, gut microbiome, middle ear microbiome, nasopharyngeal microbiome

## Abstract

Acute otitis media (AOM) is a major driver of paediatric antibiotic prescriptions. We assessed the impact of oral and topical antibiotics on middle ear, nasopharyngeal, and gut microbiome compositions, and the gut resistome, in children with AOM and ear discharge (AOMd). Fifty-eight children with AOMd and ear pain and/or fever were randomized to oral amoxicillin suspension (*n* = 31) or hydrocortisone-bacitracin-colistin eardrops (*n* = 27) for 7 days. From 57 out of 58 children, baseline, and Week-2 middle ear fluid (MEF) and nasopharyngeal (NP) samples were sequenced, along with baseline, Week-2, and Month-3 faecal samples. At baseline, the top 5 MEF genera were *Streptococcus*, *Haemophilus*, *Turicella*, *Staphylococcus* and *Alloiococcus* and NP genera *Moraxella*, *Haemophilus*, *Streptococcus*, *Corynebacterium*, and *Dolosigranulum.* At Week-2, the ear discharge had resolved in all but four children (oral *n* = 3, eardrops *n* = 1). In NP samples, the relative and absolute abundances of *Streptococcus* decreased to a greater extent after oral than eardrop treatment, but *Moraxella* and *Haemophilus* increased only following oral treatment. Neither treatment significantly altered the faecal microbiome or resistome at Week-2 and Month-3. Therefore, both treatments resolved the middle ear discharge in most children, but oral amoxicillin suspension may reduce NP *Streptococcus* more than hydrocortisone-bacitracin-colistin eardrops at the cost of potentially increasing other NP pathobionts.

## Introduction

Acute otitis media (AOM) is a leading cause for antibiotic prescriptions in children [1], affecting up to 85% of children by 3 years old, with incidence peaking between 6 and 15 months [2]. The most frequently implicated bacterial pathobionts are *Streptococcus pneumoniae*, *non-typeable Haemophilus influenzae*, *Moraxella catarrhalis, Staphylococcus aureus*, and *Streptococcus pyogenes* [3, 4].

Around 15%–20% of children with AOM present with ear discharge (AOMd) [5, 6]. Dutch guidelines recommend oral amoxicillin as the first-line treatment for children in primary care with AOMd and ear pain and/or fever, with topical antibiotics considered should ear discharge persist after 1 week [7]. This latter recommendation is supported by indirect evidence from a Dutch trial demonstrating that hydrocortisone-bacitracin-colistin eardrops are more effective than oral amoxicillin-clavulanate suspension in children with acute ear discharge and ventilation tubes [8]. In contrast, the prematurely terminated Dutch PLOTS randomized trial in 58 children with AOMd, but without ventilation tubes, found faster symptom resolution, shorter duration of ear discharge, and lower mean ear pain scores with oral amoxicillin than with hydrocortisone-bacitracin-colistin eardrops [9]. However, fewer subsequent antibiotic courses and adverse events occurred in the eardrops group [9]. An English three-armed, individually randomized trial comparing ciprofloxacin eardrops, delayed amoxicillin suspension, and immediate amoxicillin suspension suggested similar symptom resolution times across groups[10].

Antibiotic exposure may alter the microbiome compositions of various body niches. The nasopharynx, connected to the middle ear through the Eustachian tube, may act as a reservoir for bacterial pathobionts capable of invading the middle ear in specific circumstances, resulting in AOM [11, 12]. Antibiotic-induced disruption of the typically stable nasopharyngeal (NP) microbiome has been hypothesized to increase middle ear infection risk by promoting the colonization and/or outgrowth of pathobionts [13, 14]. Although often transient, NP dysbiosis – particularly reduced relative abundances (RAs) of *Corynebacterium* and *Dolosigranulum* sp. – has been associated with negative health outcomes in children, including increased viral infection risk [13, 14] and AOM [15, 16]. Antibiotics also affect the gut microbiota; early-life exposure is linked to reduced beneficial commensals (e.g., *Bifidobacterium* [17, 18] and *Lactobacillus* [17, 19]) and increased pathobionts (e.g., *Escherichia coli*) [17]. While the gut microbiome generally recovers after antibiotic treatment, even transient dysbiosis may have longer-term immunological consequences, including increased risk of developing metabolic and celiac diseases [20, 21]. Therefore, antibiotics prescribed to target putative AOM-causative bacteria may also cause collateral damage to local microbiomes and promote induction of antibiotic resistance genes (ARGs) [4].

We sequenced middle ear fluid (MEF), NP, and faecal samples of children with AOMd enrolled in the PLOTS randomized trial comparing oral amoxicillin suspension and hydrocortisone-bacitracin-colistin eardrops [9, 22]. Our aim was to evaluate and compare the impact of the eardrop and oral suspension treatments on the MEF, NP, and gut microbiome compositions at baseline, Week-2, and Month-3. We hypothesized that eardrop treatment might alter the middle ear and NP microbiomes, which are anatomically connected, but not the gut microbiome or resistome, whereas oral suspension treatment might alter all microbiomes and the gut resistome.

## Methods

### Study design

The PLOTS trial design and primary analysis results have been reported elsewhere, according to the CONSORT statement [9, 22]. The primary manuscript reported clinical endpoints and did not include any sequencing data. This paper reports on secondary analyses of sequencing data. Between December 2017 and February 2023, children aged 6 months to 12 years presenting to their general practitioner with AOMd, and ear pain or/or fever, were eligible for trial participation. Children were randomized to either oral amoxicillin suspension (Sandoz B.V., 50 mg/kg of body weight per day, divided over three doses) or hydrocortisone-bacitracin-colistin eardrops (Daleco Pharma B.V., five drops three times daily) for 7 days. Children who received antibiotics in the 2 weeks prior to screening were excluded. The study was approved by the NedMec medical ethics committee in Utrecht, The Netherlands (NedMec protocol number 17–400/G-M).

### Sample collection

MEF and NP samples were collected at baseline (prior to randomization) and at Week-2 (1 week after treatment cessation) by a trained trial physician during a home visit using a flexible applicator swab with a flocked nylon-fibre tip (ESwab, Copan Diagnostics Inc., Murrieta, CA). They were transported by the study team to the University Medical Center Utrecht (UMCU) microbiology laboratory on the day of collection or occasionally the day thereafter. Faecal samples were collected by a parent at baseline, Week-2, and Month-3, using the OMNIgene·GUT (OMR-200) system (marketed by DNA Genotek Inc., Ottawa, Canada) and mailed to the UMCU microbiology laboratory. These samples arrived in the laboratory in a median of 2 days (interquartile range: 1–5 days). Laboratory staff processed the samples on the day of receipt or the day thereafter, and stored them at −80 °C. MEF and NP samples were sequenced (16S rRNA amplicon sequencing) at the National Institute for Public Health and the Environment (RIVM, Bilthoven, the Netherlands), and faecal samples (metagenomic sequencing) at the Utrecht Sequencing Facility (USEQ, Utrecht, the Netherlands).

### 16S rRNA amplicon sequencing of NP and MEF samples

DNA extraction, 16S rRNA amplicon sequencing, and raw data processing of the NP and MEF samples were performed according to the protocol developed by Odendaal and colleagues [23]. DNA was extracted using the Mag Mini DNA isolation kit (ImmunoSource, Schilde, Belgium) based on a phenol-bead beating method. The V4 hypervariable region of the 16S rRNA gene was amplified using the 515F/806R primer pair [23, 24], and sample libraries were sequenced on the Illumina MiSeq platform (Illumina Inc., San Diego, CA, USA) using the MiSeq reagent kit v3 (2 × 300 bp) [23, 24]. The raw sequencing data were processed using the DADA2 R-package and annotated using the SILVA v138.2 reference database. The resulting amplicon sequence variant (ASV) table was manually cleaned, and ASVs were agglomerated to the genus level (further details are provided in the Methods section of Supplementary File 1).

The total 16S rRNA gene concentration in each NP and MEF sample (pg/ul) was determined via 16S rRNA qPCR using the StepOnePlus real-time PCR system (Thermo Fisher Scientific, Waltham, MA, USA) [23, 24]. Bacterial taxa concentrations were estimated by multiplying the relative abundances (RAs) of each taxon in a sample by the total bacterial load concentration of that sample. The estimated concentrations (ECs) were log_10_-transformed.

We manually grouped NP and MEF ASVs into six bacterial groups at genus-level based on the published literature about middle ear and nasopharyngeal microbiome compositions [13, 25–27] and about common AOM pathobionts [3, 4]. The commensal *Corynebacterium* and *Dolosigranulum* genera were combined into a single bacterial group called symbionts. The next four bacterial groups each consisted of a well-known AOM pathobiont genus: *Streptococcus*, *Haemophilus*, *Moraxella*, and *Turicella.* The sixth bacterial group consisted of all remaining taxa, referred to as ‘other’. NP and MEF samples were tested for respiratory tract viruses using Multiplex real-time PCR assays (Allpex respiratory assays 1, 2 and 3, Seegene, Seoul, South Korea; Methods section of Supplementary File 1).

### Metagenomic sequencing of faecal samples

DNA extraction was performed at the UMCU microbiology laboratory using the Agowa Mag DNA extraction kit (LGC genomics, Berlin, Germany). Sample libraries were prepared following Illumina’s DNA Nano library preparation protocol and sequenced using the Illumina NovaSeq 6000 with a 150 bp paired-end protocol at USEQ. Further experimental and quality control details are provided in the Methods section of Supplementary File 1.

Community profiling was performed with MetaPhlAn4 (v.4.0.6), using the ChocoPhlAn reference database (v.vOcCOVID2_CHOCOPhlAnSGB_202212), discarding reads not mapping to clade-specific markers. ARGs were profiled with ShortBRED, using markers identified from the ResFinder and Uniref90 databases (see the Methods section of Supplementary File 1). ShortBRED-Quantify was used to map the metagenomic reads to the markers and obtain the RA of each gene, expressed as reads per kilobase of reference sequence per million sample reads (RPKM).

### Statistical analysis

All microbiome composition analyses were performed in R, version 4.4.0 (The R Foundation for Statistical Consulting, Boston, MA, USA). The RAs of the top 10 taxa in MEF and NP samples were visualized, as well as the RAs and ECs of the bacterial groups. Mean α (Shannon and Inverse Simpson) and β (Bray–Curtis distances) diversities were calculated and compared by niche type (MEF versus NP), presence or absence of virus(es), whether the samples were taken pre- versus post-COVID-19 pandemic, and by study treatment (eardrops versus oral suspension). Differences in α diversities were tested with the Kruskal–Wallis rank-sum test and β diversities using PERMANOVA with 9999 permutations. Microbiome composition changes over time were tested for NP samples only, as only four children still had MEF after completing treatment. Untargeted differential abundance analyses comparing the full microbiome data at Week-2 to baseline were conducted using ANCOM-BC2 with Benjamini–Hochberg adjustment for false discovery (FDR cut-off of 0.05). Targeted analyses comparing bacterial group ECs at Week-2 compared to baseline included paired Wilcoxon signed-rank tests in children who had both baseline and Week-2 samples taken and bivariable linear regression models including all NP samples with the EC of each bacterial group as the continuous outcome and treatment group as the determinant. Two different models were fit: (1) comparing Week-2 samples from each treatment group to all baseline samples and (2) comparing Week-2 samples from the two treatment groups combined to all baseline samples. Finally, we compared the baseline characteristics of children who responded to treatment to those who did not using Kruskal–Wallis and χ^2^ tests. Responders were defined as having lower *Streptococcus* EC at Week-2 than baseline (see the Methods section of Supplementary File 1).

For faecal samples, we visualized the top 25 most abundant taxa and compared α and β diversities between treatment groups only. ARG analyses were conducted in R v4.2.1. α (Shannon) and β (Bray–Curtis) diversities of ARGs were calculated and compared between study treatments using the Wilcoxon test and PERMANOVA with 10,000 permutations, respectively. Untargeted multivariable association analyses (MaAsLin2) were used to identify bacteria and ARGs that were differentially abundant in the treatment groups comparing Week-2 to baseline, adjusting for age, sex (fixed effects), and sample ID (random effect).

## Results

### Study participants and samples

**Figure 1.**
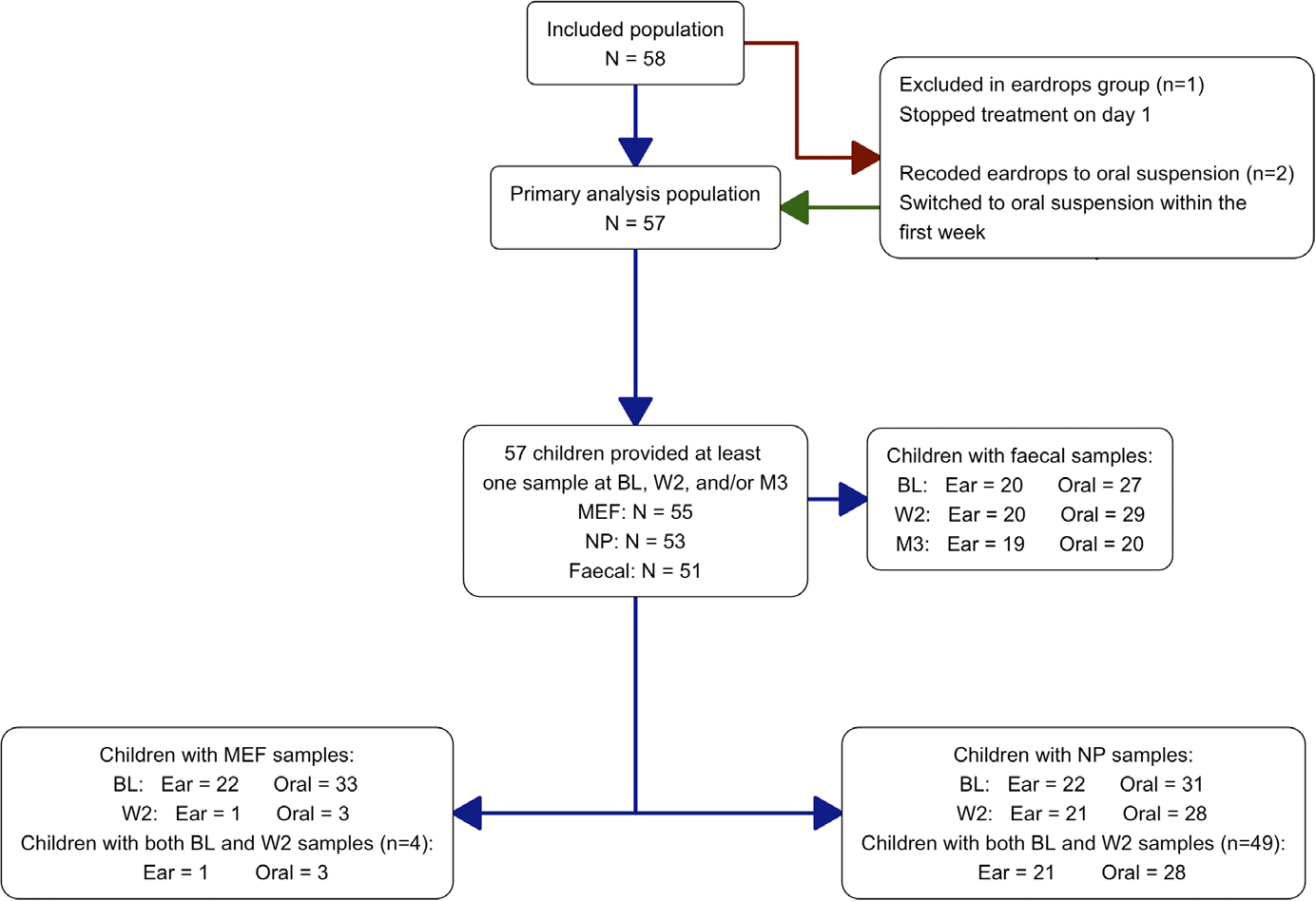
Participant and sample flow. *Note*: One child was switched from eardrops to oral treatment on day 1 because the child resisted application of the eardrops. Another child was switched from eardrops to oral treatment on day 3 because the ear pain persisted (the fever subsided). Abbreviations: BL = baseline sampling timepoint; Ear = hydrocortisone-bacitracin-colistin eardrops; MEF = middle ear fluid; M3 = month 3 sampling timepoint; NP = nasopharynx; Oral = oral amoxicillin suspension; W2 = week 2 sampling timepoint. Figure 1. long description.

Of the 58 children who participated in the PLOTS randomized trial, 57 provided at least one MEF, NP, or faecal sample during follow-up; one participant assigned to the eardrops group withdrew from the study on the day of enrolment. MEF, NP, and faecal samples from 55 out of 57 (96.5%), 53 out of 57 (93.0%), and 50 out of 57 (87.7%) children, respectively, were suitable for analysis. The participant and sample flow is shown in Figure 1. Two participants were assigned to the eardrops group but received oral antibiotic suspension early in the trial; they were analysed as part of the oral suspension group. Only four children still had ear discharge at Week-2 (oral suspension *n* = 3, eardrops *n* = 1). Baseline characteristics were well balanced between the treatment groups (Supplementary Table S.1). The mean age of the children who provided at least one MEF, NP, or faecal sample was 37.9 months (standard deviation (SD) = 27.1) and 47.4% were male (Supplementary Table S.1a). None of the MEF or NP samples tested positive for SARS-CoV-2 at baseline or Week-2 but often tested positive for other viruses (see the Results section of Supplementary File 1).

### MEF and NP microbiome compositions prior to treatment

Mean RAs of the top 10 taxa at baseline are shown in Supplementary Figure S.1a–f for the MEF and NP overall (Supplementary Figures S.1a and b), by presence or absence of any virus (Supplementary Figures S.1c and d), and by pre- or post-COVID-19 pandemic sampling (Supplementary Figures S.1e and f). Prior to treatment, *Streptococcus* dominated the MEF of children with AOMd (mean RA 50.0%), while the mean RAs of other genera were substantially lower: *Haemophilus* (12.5%), *Turicella* (11.4%), *Staphylococcus* (7.2%), *Alloiococcus* (5.7%), *Corynebacterium* (3.5%), *Moraxella* (3.5%), and *Prevotella* (1.6%) (Supplementary Figure S.1a). In the NP, the most relatively abundant genera prior to treatment were *Moraxella* (35.2%), *Haemophilus* (23.4%), *Streptococcus* (16.6%), *Corynebacterium* (10.5%), and *Dolosigranulum* (4.0%); *Turicella* and *Alloiococcus* were uncommon in this niche (Supplementary Figure S.1b). The interindividual variability is shown in Supplementary Figure S.2a and b. MEF and NP microbiome compositions were therefore distinct, further supported by significant differences in α diversities (Shannon index: *p* < 0.001, inverse Simpson index: *p* < 0.001; Supplementary Figure S.1g) and a significant β diversity (Bray–Curtis distances: PERMANOVA *p* < 0.001; Supplementary Figure S.1h). Within the same niche, however, α and β diversities did not differ by the presence or absence of at least one virus, or by pre- or post-pandemic sampling.

### Impact of treatments on the MEF and NP microbiomes

**Figure 2.**
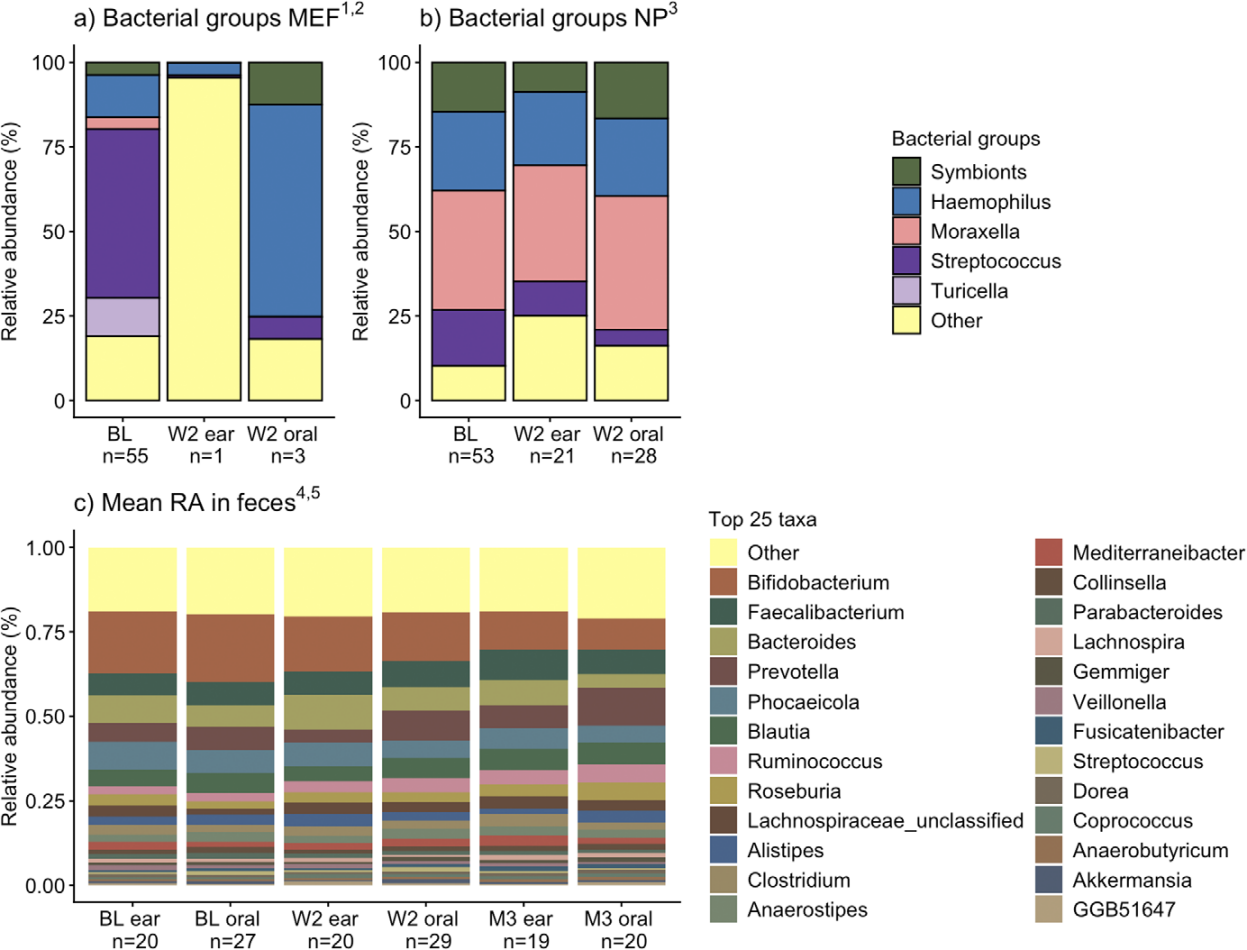
Middle ear fluid, nasopharyngeal, and gut microbiome compositions by sampling time point and treatment group in children with AOMd. *Note*: (1) The single eardrop sample at Week-2 was dominated by *Staphylococcus*, which is classified under the “other” bacterial group. (2) α diversity did not differ significantly between baseline and Week-2 MEF samples (both treatment groups combined at each time point because there were only 5 samples in total at Week-2; inverse Simpson index, Kruskal–Wallis *p* = 0.729; Shannon index, Kruskal–Wallis *p* = 0.752), whereas β diversity did differ significantly (MDS Bray–Curtis, PERMANOVA *p* = 0.020). (3) α and β diversities were not significantly different between the three groups shown in this bar graph (inverse Simpson index, Kruskal–Wallis *p* = 0.481; Shannon index, Kruskal–Wallis *p* = 0.192; and MDS Bray–Curtis, PERMANOVA *p* = 0.344). (4) Top 25 taxa at genus level. (5) α and β diversities were not significantly different between the six groups shown in this bar graph (inverse Simpson index, Kruskal–Wallis *p* = 0.917; Shannon index, Kruskal–Wallis *p* = 0.878; and MDS Bray–Curtis, PERMANOVA *p* = 0.245). Abbreviations: AOMd = acute otitis media present with ear discharge due to spontaneous perforation of the eardrum; BL = baseline sampling timepoint; Ear = hydrocortisone-bacitracin-colistin eardrops; MEF = middle ear fluid; MDS = multidimensional scaling; NP = nasopharynx; M3 = month 3 sampling timepoint; Oral = oral amoxicillin suspension; RA = relative abundance; W2 = week 2 sampling timepoint. Figure 2. long description.

The mean RAs of bacterial groups in the MEF and NP niches at baseline and Week-2 by treatment group are shown in Figure 2a,b, and the top 10 taxa in Supplementary Figure S.2c and d. In NP samples, mean RA of *Streptococcus* decreased more effectively after oral suspension than eardrop treatment: 16.6% at baseline versus 4.8% (oral) and 10.1% (eardrops) at Week-2 (Figure 2b, *p* = 0.001). In both niches and treatment groups, mean α diversity indexes remained similar over time. The β diversity of MEF samples compared for baseline to Week-2 showed a statistically significant difference (*p* = 0.020), but this was not the case for NP samples (*p* = 0.344).

**Figure 3.**
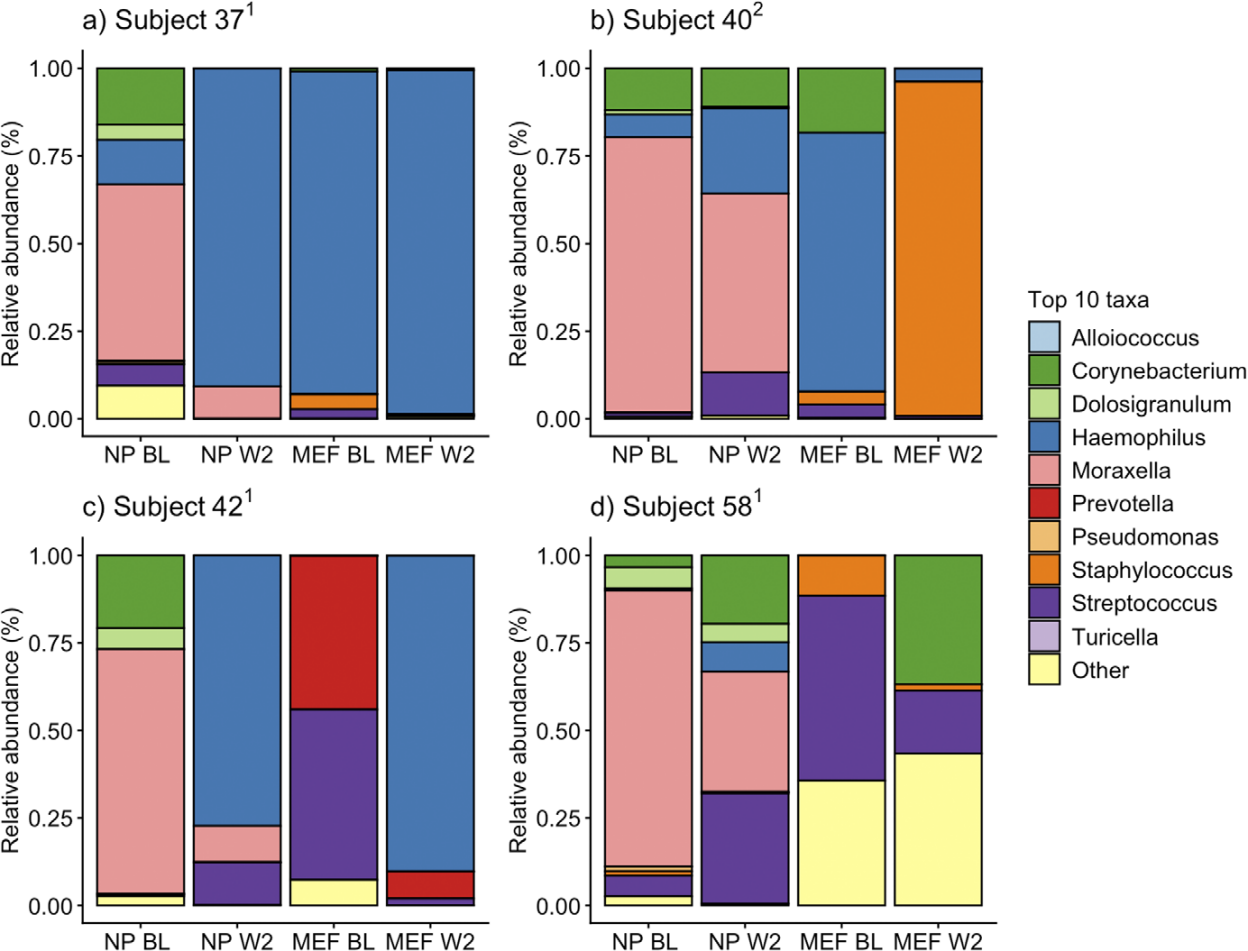
Individual MEF composition of the four children who still had ear discharge at Week-2. *Note*: (1) Received oral suspension. (2) Received eardrops. Abbreviations: BL = baseline sampling timepoint; MEF = middle ear fluid samples; NP = nasopharyngeal samples; W2 = week 2 sampling timepoint. Figure 3. long description.

Only four children still had MEF at Week-2 (Figure 3a–d). In two of these children, *Haemophilus* dominated the MEF samples at baseline, and *Haemophilus* or *Staphylococcus* after treatment. The MEF of the two other children contained high RAs of *Streptococcus*, *Prevotella*, and mixed bacteria at baseline and shifted towards *Haemophilus* domination or mixed bacteria at Week-2. These data suggest that treatment failure may be associated with having high abundance of AOMd-causing organisms other than *Streptococcus* in the middle ear.

The ANCOM-BC2 differential abundance analysis identified a decrease in the genus *Streptococcus* from baseline to Week-2 in the NP samples, with a significant reduction in the mean RA after oral suspension treatment (16.6% to 4.8%; *p* = 0.001), and a non-significant reduction after eardrop treatment (16.6% to 10.1%; *p* = 0.264). The family Neisseriaceae was also identified as differentially lower after oral suspension, although we considered the low mean RAs in the comparison groups as clinically insignificant (0.88% to 0.20%; *p* = 0.015).

**Figure 4.**
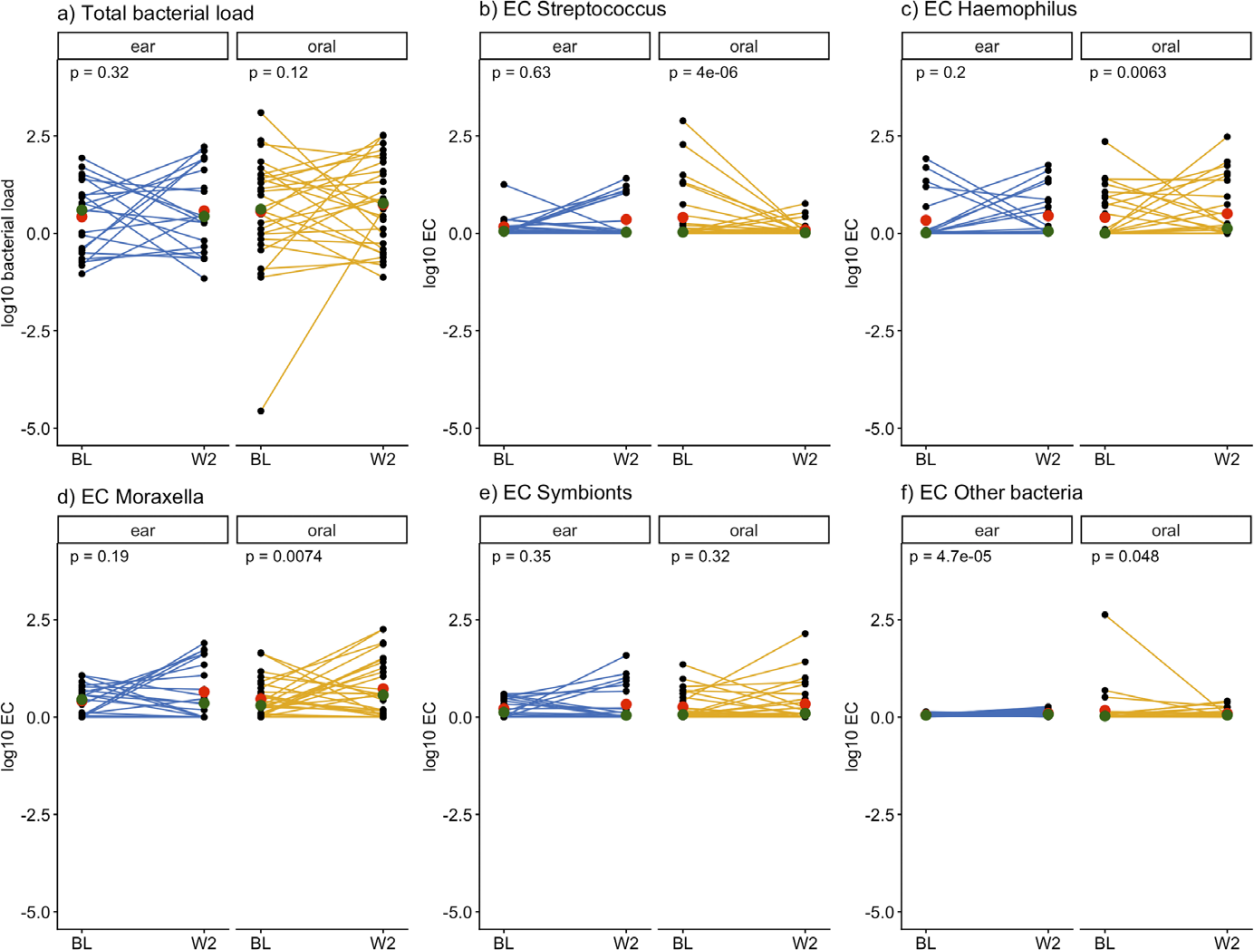
Paired analyses of total bacterial load and log_10_ estimated concentrations of bacterial groups in nasopharyngeal samples before and after eardrop or oral suspension treatment in children with AOMd. *Note*: The green dot represents the median of samples, and the red dot represents the mean. Differences in mean ECs were tested using Wilcoxon signed-rank test for paired samples. N = 49 children with both baseline and Week-2 samples; *n* = 21 ear and *n* = 28 oral antibiotics. Abbreviations: AOMd = acute otitis media present with ear discharge due to spontaneous perforation of the eardrum; BL = baseline sampling timepoint; Ear = hydrocortisone-bacitracin-colistin eardrops; EC = log10 estimated concentration in pg/ul; Oral = oral amoxicillin; W2 = week 2 sampling timepoint. Figure 4. long description.

Forty-nine children with both baseline and Week-2 NP samples were included in paired analyses of total bacterial load and ECs of bacterial groups at Week-2 compared to baseline (Figure 4). The mean total NP bacterial load was not significantly different over time in both treatment groups, although interindividual variability was high (Figure 4a). *Streptococcus* ECs and EC changes varied greatly between individuals; however, a general trend of decreasing *Streptococcus* EC in samples was observed in the oral suspension group (*p* < 0.001; Figure 4b). The mean *Haemophilus* ECs and *Moraxella* ECs slightly increased during both treatments, reaching significance in the oral suspension group only (*p* = 0.006 and *p* = 0.007, respectively; Figure 4c,d). The mean symbionts ECs remained unchanged in both groups (Figure 4e). In summary, NP *Streptococcus* EC decreased to a greater extent in the oral suspension than in the eardrops group, while *Haemophilus* and *Moraxella* ECs tended to increase in that group.

### Bivariable linear regression models of bacterial group ECs in NP samples

In bivariable linear regression models, total NP bacterial load was similar between time points and treatment groups (Table 1). NP *Streptococcus* ECs declined between baseline and Week-2 in the oral suspension group, although not statistically significantly (coefficient −0.22, 95% CI –0.45 to 0.01, *p* = 0.063). The NP *Streptococcus* EC showed a steeper decline in children who were treated with oral suspension compared to eardrops (−0.27, −0.48 to –0.05, *p* = 0.016). The NP *Moraxella* EC showed an increasing trend between baseline and Week-2 in the oral suspension group (0.25, −0.03 to 0.53, *p* = 0.079). The NP EC of symbionts remained stable in both groups. These results align with the results of the paired analyses described earlier.

**Table 1.** Bivariable linear regression models with estimated concentration of one bacterial group in nasopharyngeal samples from children with AOMd^a^ as the outcome of each model Table 1. long description.

Outcome	Determinant^b^	Coefficient (95% CI)	*p*-value
Total bacterial load	W2 ear vs. baseline all	0.03 (−0.57, 0.62)	0.927
	W2 oral vs. baseline all	0.19 (−0.35, 0.73)	0.494
	W2 oral vs. W2 ear	0.16 (−0.47, 0.79)	0.615
EC log10 Streptococcus	W2 ear vs. baseline all	0.05 (−0.21, 0.30)	0.713
	W2 oral vs. baseline all	−0.22 (−0.45, 0.01)	0.063
	W2 oral vs. W2 ear	−0.27 (−0.48, −0.05)	**0.016**
EC log10 Haemophilus	W2 ear vs. baseline all	0.04 (−0.30, 0.38)	0.814
	W2 oral vs. baseline all	0.10 (−0.21, 0.40)	0.537
	W2 oral vs. W2 ear	0.06 (−0.34, 0.45)	0.776
EC log10 Moraxella	W2 ear vs. baseline all	0.17 (−0.14, 0.48)	0.277
	W2 oral vs. baseline all	0.25 (−0.03, 0.53)	0.079
	W2 oral vs. W2 ear	0.08 (−0.35, 0.51)	0.707
EC log10 Symbionts	W2 ear vs. baseline all	0.08 (−0.13, 0.29)	0.437
	W2 oral vs. baseline all	0.09 (−0.10, 0.28)	0.346
	W2 oral vs. W2 ear	0.01 (−0.28, 0.30)	0.955
EC log10 Other	W2 ear vs. baseline all	−0.02 (−0.16, 0.12)	0.751
	W2 oral vs. baseline all	−0.03 (−0.15, 0.10)	0.695
	W2 oral vs. W2 ear	−0.003 (−0.06, 0.06)	0.926

a N = 53 children with baseline samples (BL ear = 22, BL oral = 31), and N = 49 children with samples at Week-22 (W2 ear = 21, W2 oral = 28).

b Two regression models: (1) the Week-2 samples in each treatment group are compared to all baseline samples and (2) the Week-2 samples are compared to each other, eardrops vs. oral suspension.

Abbreviations: AOMd = acute otitis media present with ear discharge due to spontaneous perforation of the eardrum; CI = Confidence interval; Ear = hydrocortisone-bacitracin-colistin eardrops; EC = log10 estimated concentration in pg./ul; Oral = oral amoxicillin; W2 = week 2 sampling timepoint.

### NP microbiomes and clinical characteristics of treatment responders to non-responders

No differences were observed in the total NP bacterial load between responders (mean = 0.56 log_10_ pg/ul) and non-responders (mean = 0.65, *p* = 0.338) when data from both treatment groups and across sampling time points were combined. Total NP bacterial load increased between baseline and Week-2 samples among the non-responders (baseline log_10_ mean = −0.03 and Week-2 log_10_ mean = 1.32, *p* = 0.003), but not in the responders (*p* = 0.220; see the Results section of Supplementary File 1).

### Impact of treatments on the gut microbiome composition and gut resistome

The gut microbiome composition was highly diverse at all time points (Figure 2c). In the baseline samples, the most abundant genera prior to treatment were *Bifidobacterium* (mean RA 19.3%), *Phocaeicola* (7.4%), *Bacteroides* (7.2%), *Faecalibacterium* (6.7%), *Prevotella* (6.2%), *Blautia* (5.5%), and *Ruminococcus* (2.4%). α and β diversities were not different between treatment groups and across timepoints.

A total of 117 ARGs were identified in the faecal samples with tetracycline resistance genes the most abundant class and tet(Q)_3 the most abundant ARG (Supplementary Figure S.3a; Results section of Supplementary File 1). However, MaAsLin2 analyses did not identify any ARGs to be significantly over- or underrepresented across the treatment groups. The α-diversity (Shannon) and β-diversity (Bray–Curtis) of the ARGs were similar between the treatment groups at baseline, Week-2, and Month-3 (Supplementary Figures S.3b-d), except for a significantly higher Shannon diversity in the oral group at Week-2 (*p* = 0.042). However, by Month-3, the Shannon diversity was similar between the treatment groups (*p* = 0.667). No statistically significant differences were observed in total ARG RAs between the treatment groups at baseline, Week-2, or Month-3 (Supplementary Figure S.3e).

Sensitivity analyses for NP and gut microbiomes excluding children that received additional antibiotic treatment (in any formulation) during follow-up yielded similar results (Supplementary File 2).

## Discussion

We compared MEF, NP, and gut microbiome compositions of children with AOMd who were randomly assigned to oral amoxicillin suspension or hydrocortisone-bacitracin-colistin eardrops treatment groups. Prior to treatment, the MEF samples were dominated by *Streptococcus*, whereas the NP samples were more diverse. Ear discharge was resolved in all but four children by Week-2 (oral suspension *n* = 3, eardrops *n* = 1). In the cases with persistent ear discharge, baseline and Week-2 MEF samples were not dominated by *Streptococcus* but by *Haemophilus* or *Staphylococcus*, or contained mixed bacteria. In the NP samples, oral suspension led to a greater reduction in *Streptococcus* RA and EC compared to eardrops, but *Haemophilus* and *Moraxella* ECs increased following oral suspension treatment. This represents genuine outgrowth because the EC findings are quantitative, not relative. Neither treatment affected NP symbionts or the gut microbiome or resistome compositions.

The 10 most relatively abundant taxa in our MEF samples were (in order of decreasing mean RA) *Streptococcus, Haemophilus, Turicella, Staphylococcus, Alloiococcus, Corynebacterium, Moraxella, Prevotella, Finegoldia*, and *Escherichia-Shigella.* This is similar to other studies in children with AOMd [28, 29]. Although the nasopharynx is often used as a proxy for the middle ear [27, 30], our results suggest substantial differences between these niches, consistent with a previous systematic review indicating that NP samples do not provide a good proxy for the middle ear [30]. However, NP samples are of interest in their own right due to the anatomical connection with the middle ear.

*Corynebacterium* and *Dolosigranulum* enrichment in the NP has been associated with reduced pathobiont colonization and may be protective against AOM [11, 31]. *Corynebacterium* and *Dolosigranulum* typically comprise 13%–33% [32–34] and 2%–15% [32–35] of the NP microbiome, respectively, with higher RAs observed in children [34, 35]. In the PLOTS study, their combined RAs were 14.5% at baseline and 13.2% after treatment. In one study, children with higher RAs of *Corynebacterium* and *Dolosigranulum* in the NP were less likely to have AOM [11]. Furthermore, higher RAs of *Corynebacterium* and *Dolosigranulum* was negatively associated with colonization by *S. pneumoniae*, *H. influenzae*, and *M. catarrhalis* [11]. In our study, neither treatment negatively affected the nasopharyngeal *Corynebacterium* and *Dolosigranulum* abundances.

While recent systematic reviews suggest that systemic antibiotics can have lasting effects on the gut microbiome, findings are often inconsistent and data on children are limited [18, 36]. In contrast, we found no evidence that a 7-day course of either oral suspension or eardrops significantly disrupted commensal bacteria in the gut microbiome or increased the gut resistome.

In the Netherlands, oral amoxicillin suspension is the first-line treatment for AOM if antibiotics are indicated, while hydrocortisone-bacitracin-colistin eardrops may be used should ear discharge persist beyond 1 week [7]. Amoxicillin is a broad-spectrum β-lactam antibiotic [12, 37]. Hydrocortisone-bacitracin-colistin eardrops contain two antibiotics: bacitracin, primarily targeting Gram-positive bacteria, and colistin, which targets Gram-negative bacteria [38]. In our study, oral amoxicillin suspension reduced *Streptococcus* species in the NP more effectively than eardrops. We could not assess this in the middle ear because only four children still had ear discharge after treatment completion, and because the NP microbiome was not a good proxy for the MEF microbiome. We also found that *Haemophilus* and *Moraxella* increased in the NP after amoxicillin exposure. This aligns with the existing literature reports that the effectiveness of amoxicillin against *H. influenza* and *M. catarrhalis* is often compromised due to β-lactamase production by some strains [12, 39, 40]. The observed increases may be due to the selective pressure of amoxicillin: By effectively reducing *Streptococcus*, the outgrowth of *Haemophilus* and *Moraxella* may be facilitated. While less effective against *Haemophilus* and *Moraxella* compared to *Streptococcus*, amoxicillin is generally effective in resolving clinical AOM symptoms [39] and, in the PLOTS trial, at least as effective as hydrocortisone-bacitracin-colistin eardrops [9]. In our study, all children with persistent ear discharge at Week-2 had resolved symptoms by Month-3. Furthermore, certain *Streptococcus* species, including *S. pneumoniae* and viridans group streptococci, are known to acquire antibiotic resistance [40]. Consistently, children in our study who continued to have increased *Streptococcus* in the NP by Week-2 had more prior AOMd episodes, suggesting that previous antibiotic use may promote acquisition of resistant *Streptococcus* strains. Although clinical symptoms did resolve by Month-3, future trials should consider the underlying bacterial profiles of children with AOM to ensure antibiotics administered target the intended pathobionts.

This is the first randomized trial comparing oral amoxicillin suspension and hydrocortisone-bacitracin-colistin eardrops on the middle ear, nasopharynx, and gut microbiome compositions in children with AOMd. The trial was limited by early termination due to slow recruitment, enrolling 58 children instead of the planned 350. However, the original sample size calculations were based on the primary trial outcome, not the outcome of the exploratory analyses presented here, and we did uncover several statistically significant and clinically relevant results. Second, antibiotic use within 2 weeks prior to randomization was an exclusion criterion; however, cumulative prior antibiotic exposure data were not available. Third, samples were obtained at 2 weeks and 3 months (faecal samples only) post randomization, but not immediately following treatment completion (day-7), potentially missing transient – but less clinically relevant – effects. Fourth, all children had AOMd and received antibiotics; placebo or no-treatment groups were not included for ethical reasons, leaving us unable to investigate children with AOMd in the absence of antibiotic treatment.

In conclusion, both treatments resolved the AOMd in most children. However, oral amoxicillin suspension may reduce nasopharyngeal *Streptococcus* abundance more effectively than hydrocortisone-bacitracin-colistin eardrops, while increasing *Haemophilus* and *Moraxella* abundance. Neither treatment appears to adversely affect nasopharyngeal or gut commensals, or the gut resistome. There is a need for antibiotics that are highly effective against all three key NP pathobionts, and *Dolosigranulum* and *Corynebacterium* may hold promise as future live biotherapeutic agents. We recommend that future intervention trials assess treatment effects on relevant microbiomes, enabling both beneficial outcomes and unintended off-target microbiological effects to be clearly quantified.

## Supporting information

10.1017/S0950268826101836.sm001 Claus et al. supplementary material Claus et al. supplementary material

## Data Availability

The meta data is stored in the digital archive DataverseNL, https://dataverse.nl/dataset.xhtml?persistentId=doi:10.34894/J4KPP1. Raw sequencing reads are available on the European Nucleotide Archive (ENA), under accession number PRJEB94155. All code used in this manuscript can be found at https://github.com/rosssmcinnes/ and https://github.com/juanaclaus/PLOTS_microbiome.git.

## Supplementary material

The supplementary material for this article can be found at http://doi.org/10.1017/S0950268826101836.

## Long descriptions

### Figure 1. Long description

Abbreviations: BL = baseline sampling timepoint; Ear = hydrocortisone-bacitracin-colistin eardrops; MEF = Middle ear fluid; M3 = month 3 sampling timepoint; NP=Nasopharynx; Oral = oral amoxicillin suspension; W2 = week 2 sampling timepoint. One child was switched from eardrops to oral treatment on Day 1 because the child resisted application of the eardrops. Another child was switched from eardrops to oral treatment on Day 3 because the ear pain persisted (the fever subsided).

Navigate back to Figure 1..

### Figure 2. Long description

Abbreviations: AOMd = acute otitis media present with ear discharge due to spontaneous perforation of the eardrum; BL = baseline sampling timepoint; Ear = hydrocortisone-bacitracin-colistin eardrops; MEF = Middle ear fluid; MDS = Multidimensional scaling; NP=Nasopharynx; M3 = month 3 sampling timepoint; Oral = oral amoxicillin suspension; RA = Relative abundance; W2 = week 2 sampling timepoint. 1. The single eardrop sample at Week-2 was dominated by Staphylococcus, which is classified under the “other” bacterial group. 2. Alpha diversities did not differ significantly between baseline and Week-2 MEF samples (both treatment groups combined at each time point because there were only 5 samples in total at Week-2; Inverse Simpson index, Kruskal Wallis p = 0.729; Shannon index, Kruskal Wallis p = 0.752), whereas beta diversity did differ significantly (MDS Bray-Curtis, PERMANOVA p = 0.020). 3. Alpha and beta diversities were not significantly different between the three groups shown in this bar graph (Inverse Simpson index, Kruskal Wallis p = 0.481; Shannon index, Kruskal Wallis p = 0.192; and MDS Bray-Curtis, PERMANOVA p = 0.344). 4. Top 25 taxa at genus level. 5. Alpha and beta diversities were not significantly different between the six groups shown in this bar graph (Inverse Simpson index, Kruskal Wallis p = 0.917; Shannon index, Kruskal Wallis p = 0.878; and MDS Bray-Curtis, PERMANOVA p = 0.245).

Navigate back to Figure 2..

### Figure 3. Long description

Abbreviations: BL = baseline sampling timepoint; MEF = Middle ear fluid samples; NP=Nasopharyngeal samples; W2 = week 2 sampling timepoint. 1. Received oral suspension. 2. Received eardrops.

Navigate back to Figure 3..

### Figure 4. Long description

Abbreviations: AOMd = acute otitis media present with ear discharge due to spontaneous perforation of the eardrum; BL = baseline sampling timepoint; Ear = hydrocortisone-bacitracin-colistin eardrops; EC = log10 estimated concentration in pg./ul; Oral = oral amoxicillin; W2 = week 2 sampling timepoint. The green dot represents the median of samples, and the red dot represents the mean. Differences in mean ECs were tested using Wilcoxon signed-rank test for paired samples. N = 49 children with both baseline and Week-2 samples; n = 21 ear and n = 28 oral antibiotics.

Navigate back to Figure 4..

### Table 1. Long description

Abbreviations: AOMd = acute otitis media present with ear discharge due to spontaneous perforation of the eardrum; CI = Confidence interval; Ear = hydrocortisone-bacitracin-colistin eardrops; EC = log10 estimated concentration in pg/ul; Oral = oral amoxicillin; W2 = week 2 sampling timepoint. 1. N = 53 children with baseline samples (BL ear = 22, BL oral = 31), and N = 49 children with samples at Week-22 (W2 ear = 21, W2 oral = 28). 2. Two regression models: (1) the Week-2 samples in each treatment group are compared to all baseline samples and (2) the Week-2 samples are compared to each other, eardrops vs. oral suspension.

Navigate back to Table 1..
