# Supplementary material for: The impact of topical or oral antibiotics in children with acute otitis media on their middle ear, nasopharyngeal, and gut microbiomes
Source: Epidemiol Infect. 2026 Jun 23;154:e94. doi: 10.1017/S0950268826101836 (PMC13366364; doi:10.1017/S0950268826101836)
Supplement: Claus et al. supplementary material [file S0950268826101836sup001.zip › 260225_Supplementary Figure S1.docx]

**Figure S1: Baseline middle ear fluid and nasopharyngeal microbiome compositions and diversity in children with AOMd prior to antibiotic treatment^1^**

**
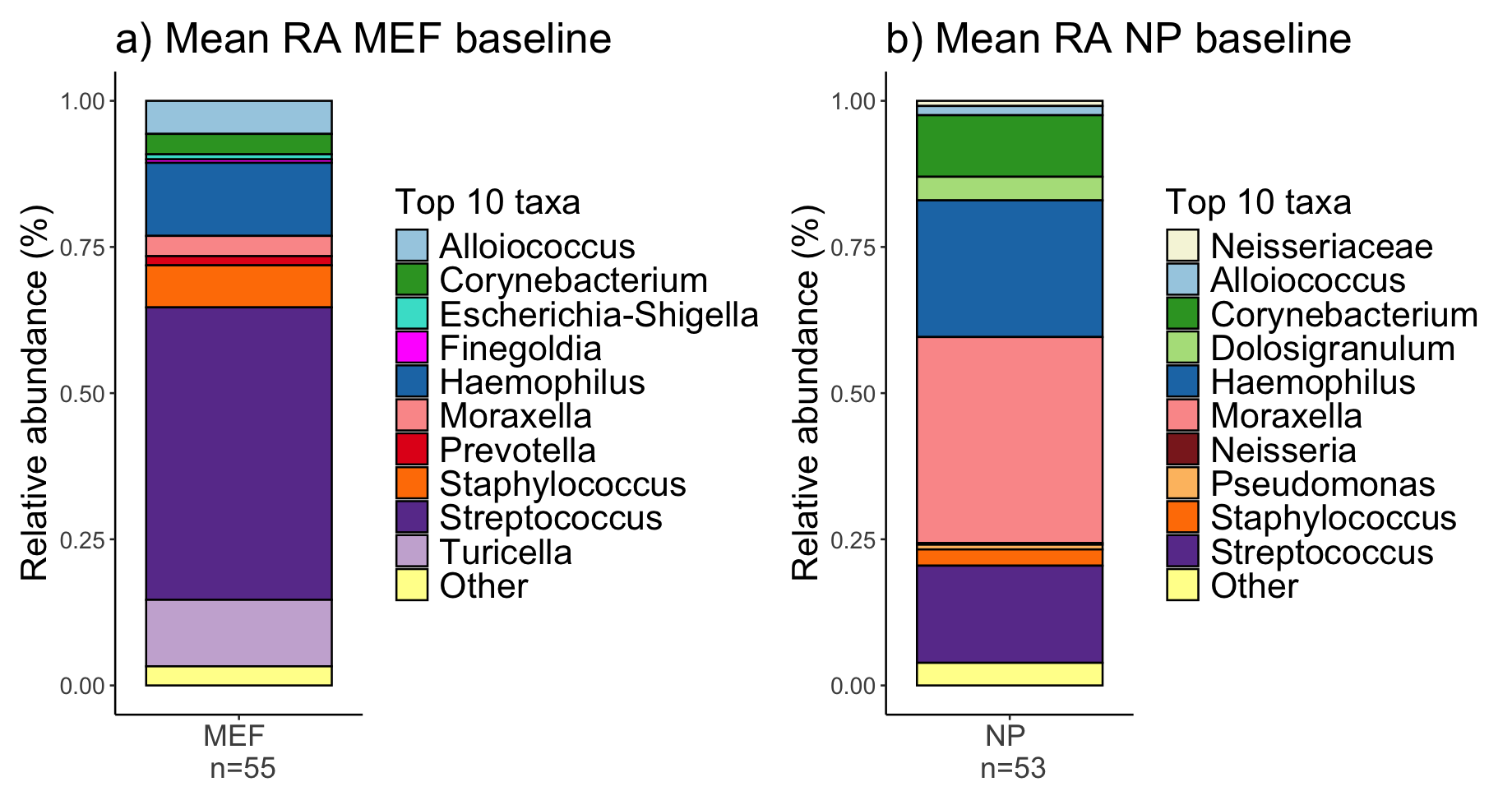
**

**
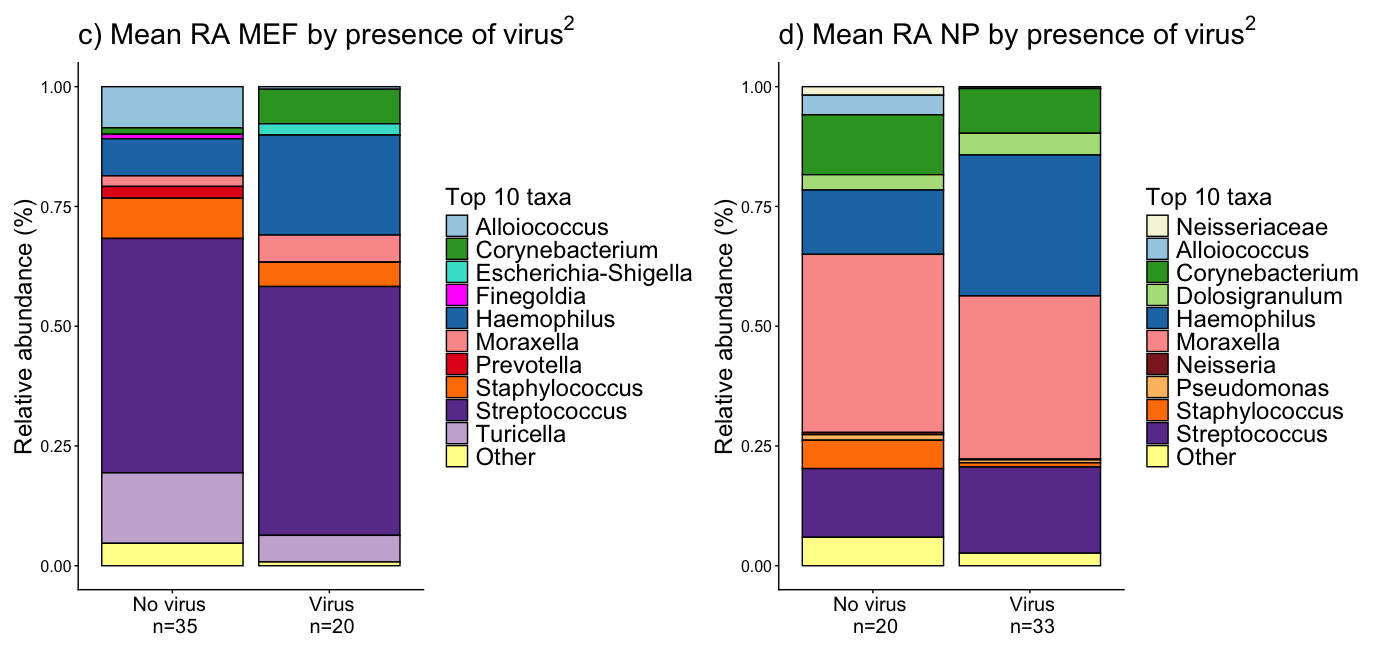
**

**
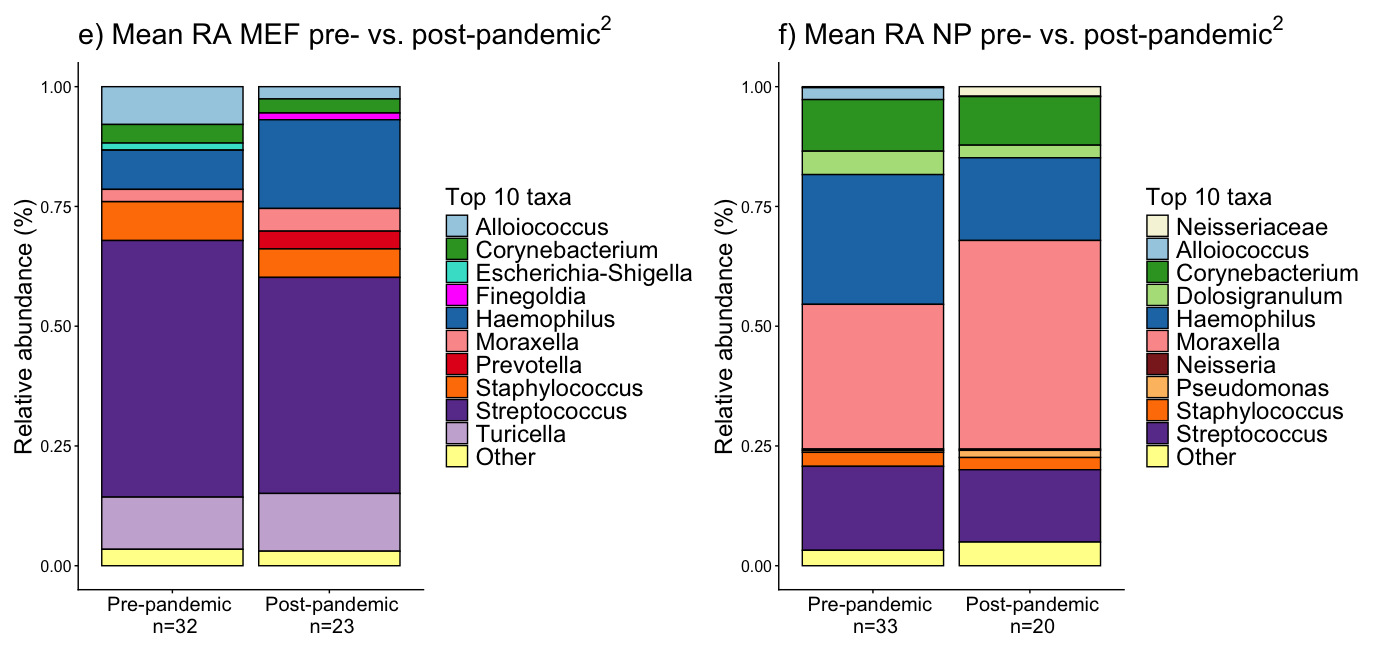
**


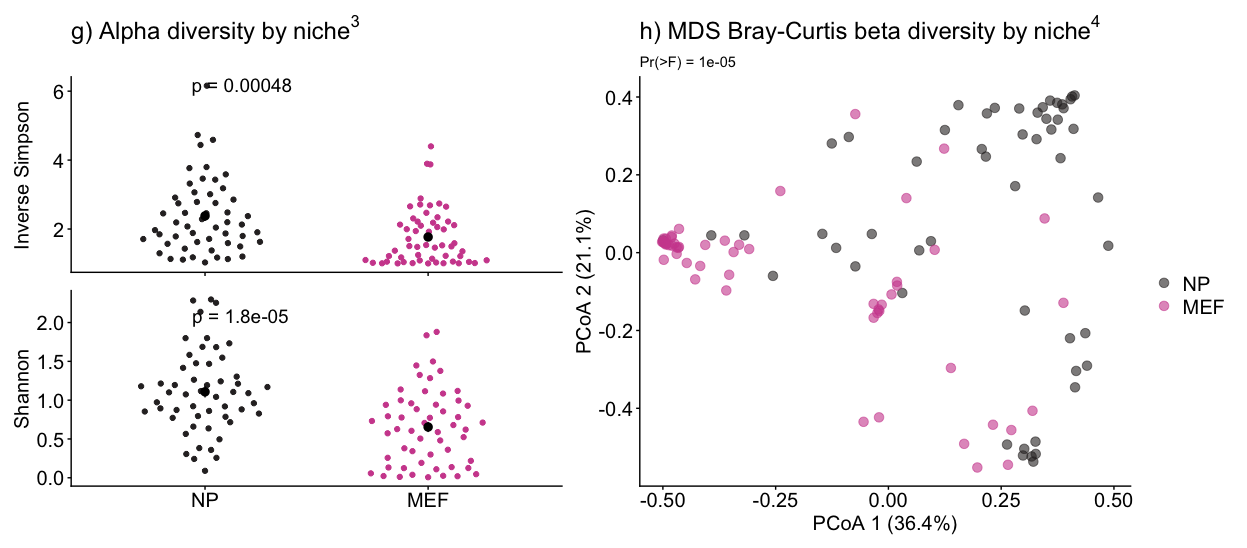


Abbreviations: AOMd= acute otitis media present with ear discharge due to spontaneous perforation of the eardrum; MDS=Multidimensional scaling; MEF=Middle ear fluid samples; No virus= the sample tested negative for all viruses included in the viral panel; NP=Nasopharyngeal samples; Oral=oral amoxicillin suspension; PCA= Principal Coordinate Analysis; RA= relative abundance; Virus= the sample tested positive for at least one virus included in the viral panel.

1. For analysis of the baseline samples, the oral suspension and eardrop groups were combined.
2. Overall mean alpha diversity tested with Wilcoxon test. Beta diversities were compared using PERMANOVA, with 9999 permutations. Alpha and beta diversities between groups were not statistically significant.
3. Overall mean alpha diversity tested with Wilcoxon test.
4. Beta diversities were compared using PERMANOVA, with 9999 permutations.
