# Supplementary material for: The impact of topical or oral antibiotics in children with acute otitis media on their middle ear, nasopharyngeal, and gut microbiomes
Source: Epidemiol Infect. 2026 Jun 23;154:e94. doi: 10.1017/S0950268826101836 (PMC13366364; doi:10.1017/S0950268826101836)
Supplement: Claus et al. supplementary material [file S0950268826101836sup001.zip › 260225_Supplementary Figure S2.docx]

**Figure S2: Middle ear fluid and nasopharyngeal relative abundance of top 10 taxa at baseline and Week-2**

**
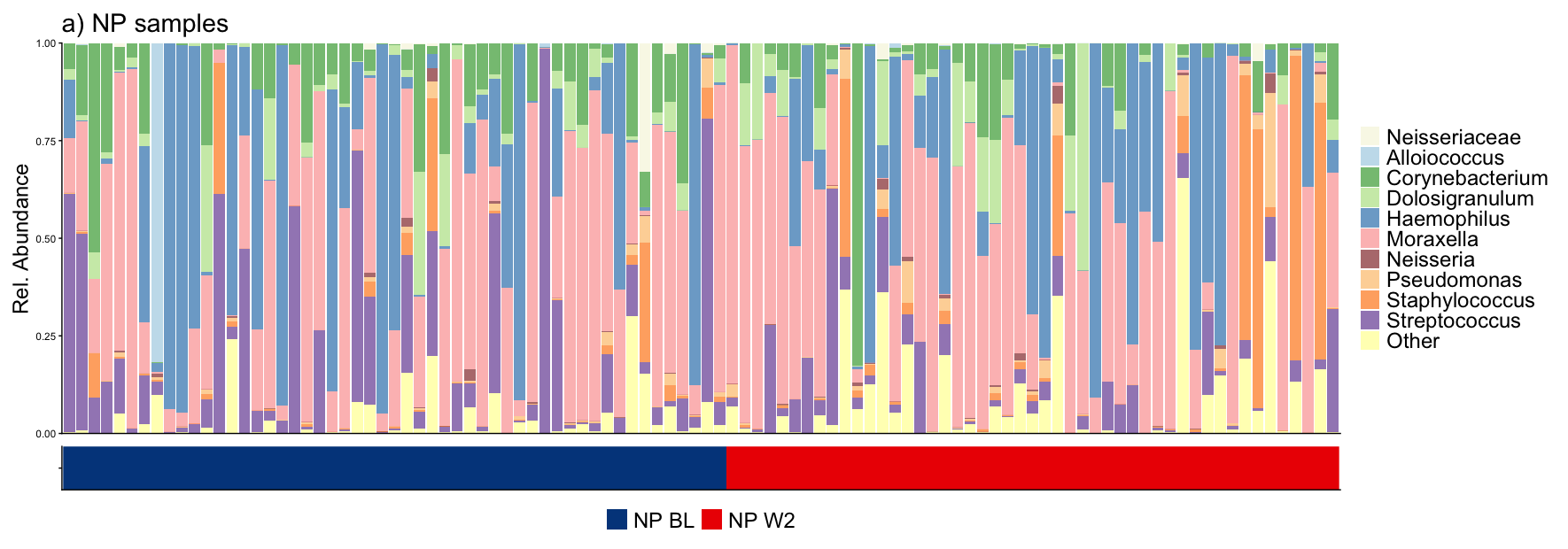
**

**
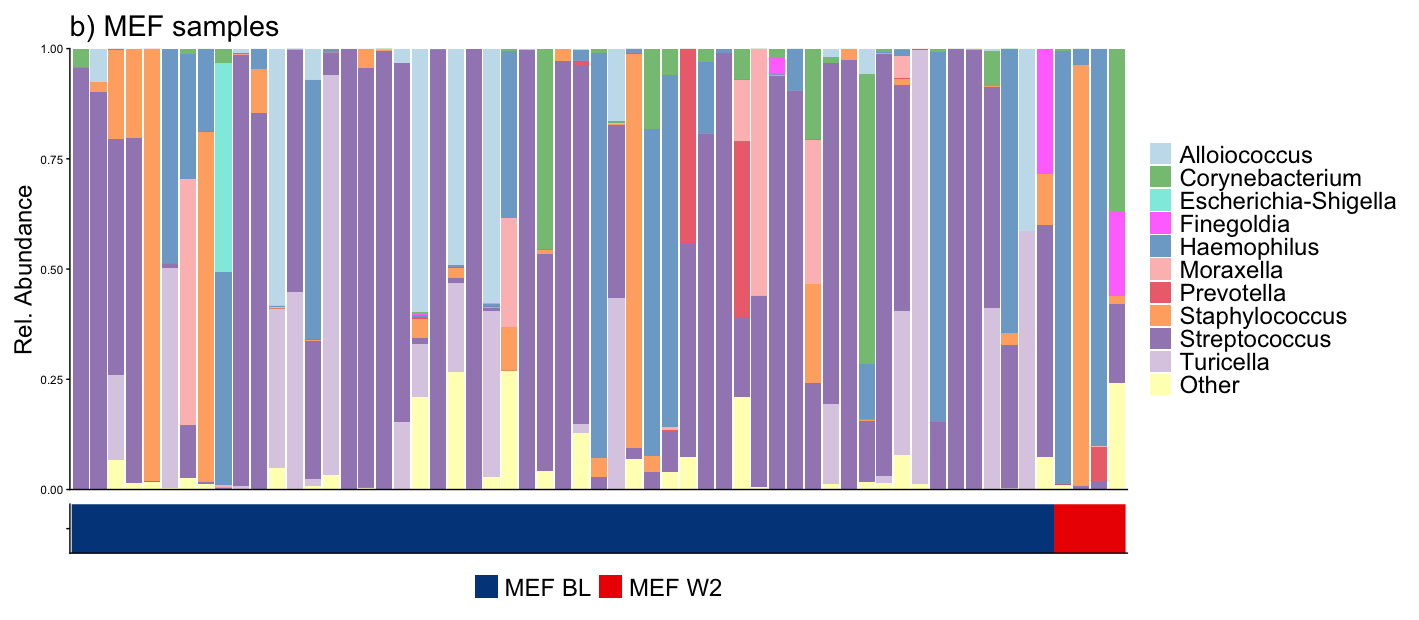
**


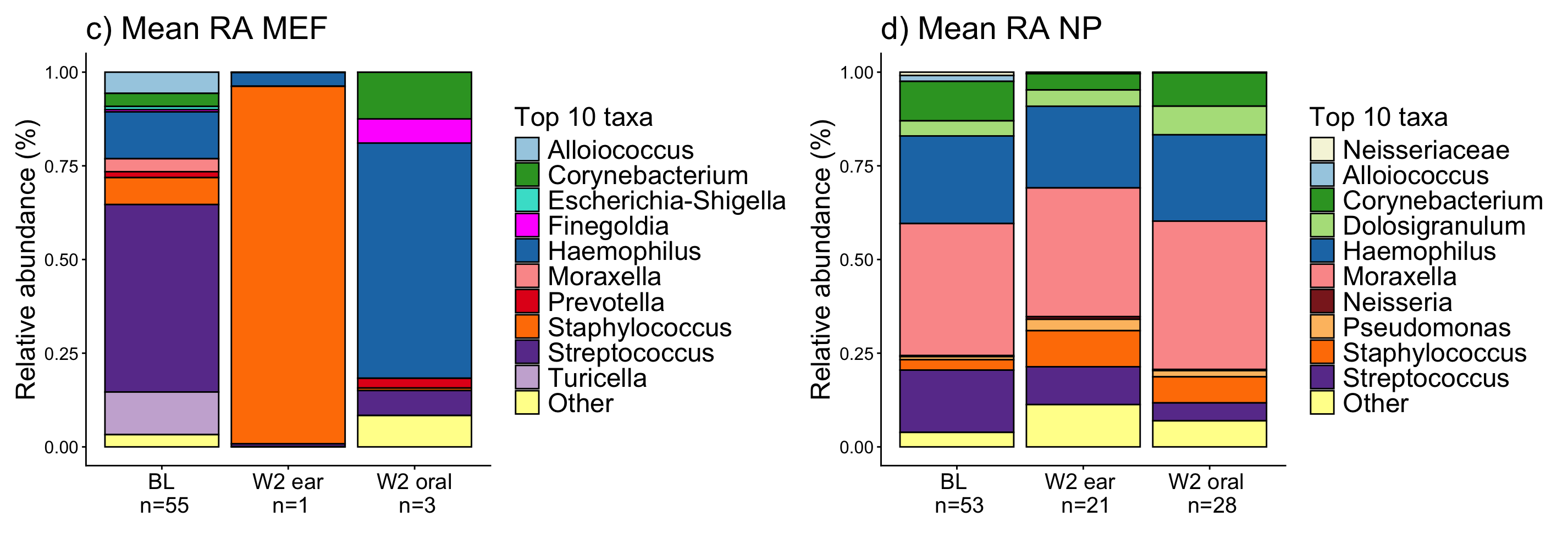


Abbreviations: BL=baseline sampling timepoint; Ear=hydrocortisone-bacitracin-colistin eardrops; MEF=Middle ear fluid samples; NP=Nasopharyngeal samples; Oral=oral amoxicillin suspension; RA=Relative abundance; W2=week 2 sampling timepoint.
