# Supplementary material for: The impact of topical or oral antibiotics in children with acute otitis media on their middle ear, nasopharyngeal, and gut microbiomes
Source: Epidemiol Infect. 2026 Jun 23;154:e94. doi: 10.1017/S0950268826101836 (PMC13366364; doi:10.1017/S0950268826101836)
Supplement: Claus et al. supplementary material [file S0950268826101836sup001.zip › 260225_Supplementary Figure S3.docx]

**Figure S3: The impact of antibiotic treatments on antibiotic resistance genes present within the gut microbiome of children with AOMd.**

a) The 20 most abundant antibiotic resistance genes in gut microbiome samples^1^


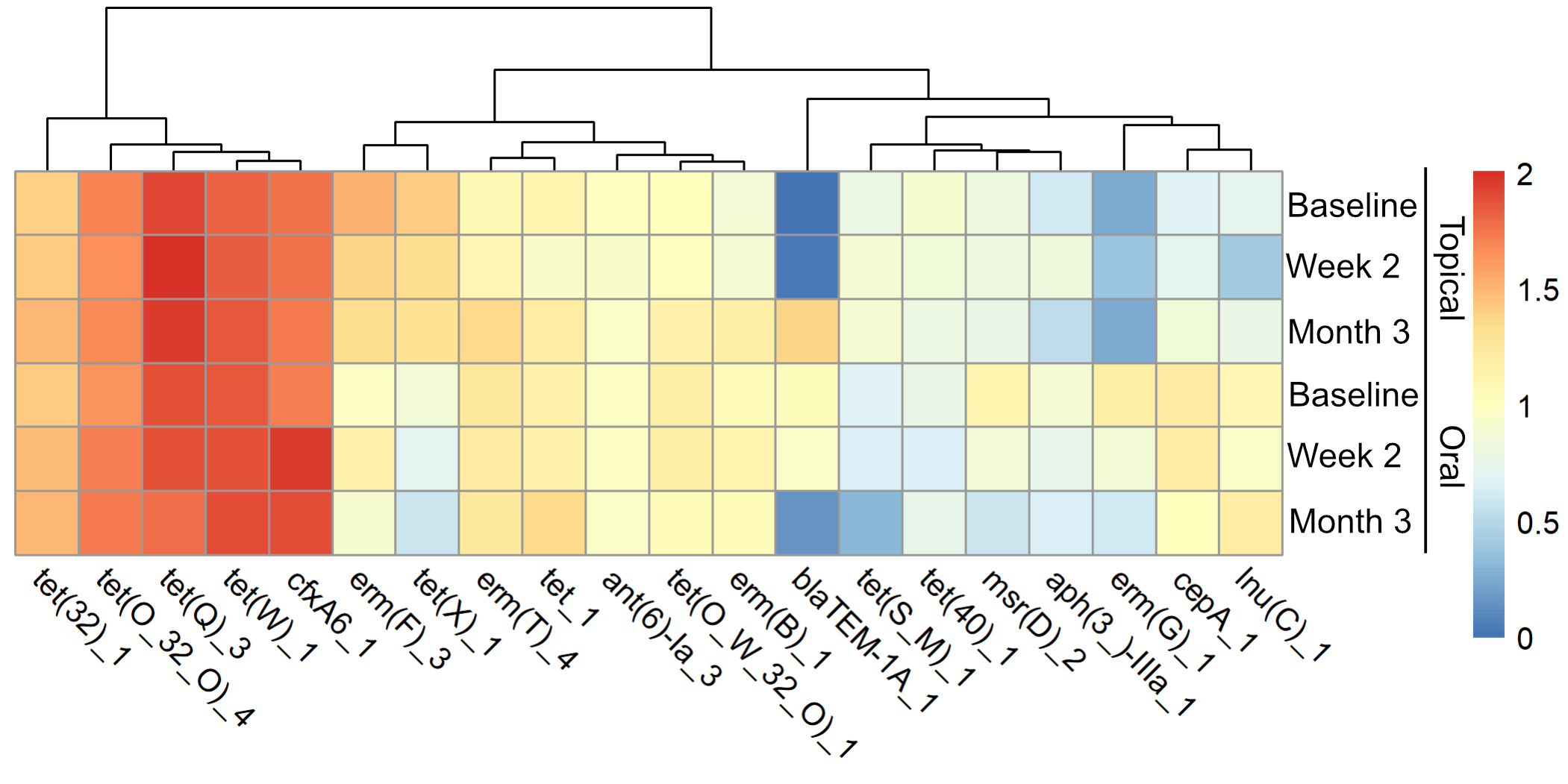


b) Alpha diversity of antibiotic resistance genes in gut microbiome sample^2^

**
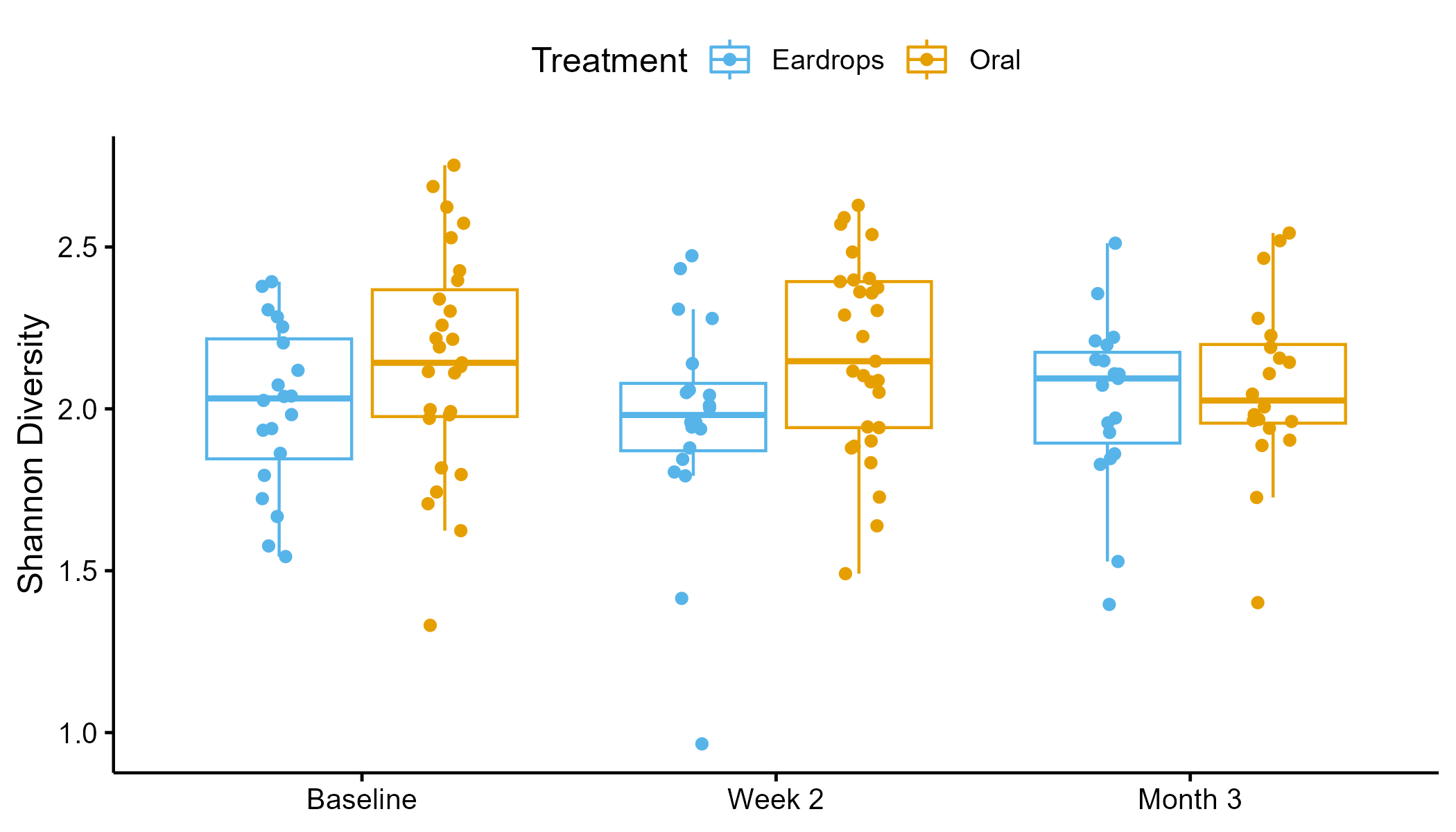
**

c) Beta diversity of antibiotic resistance genes in gut microbiome samples prior to treatment^3^


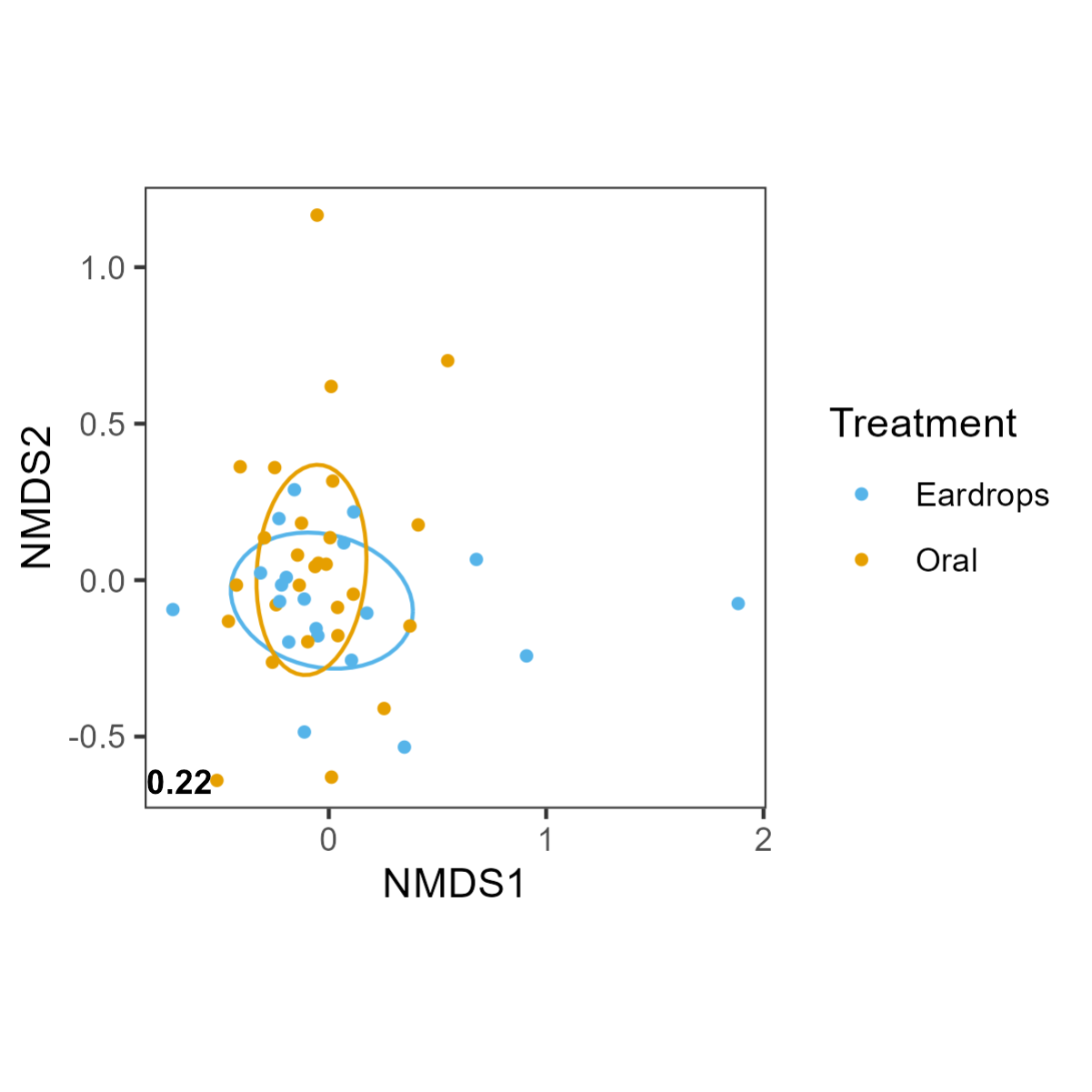


d) Beta diversity of antibiotic resistance genes in gut microbiome samples one-week post-treatment^3^


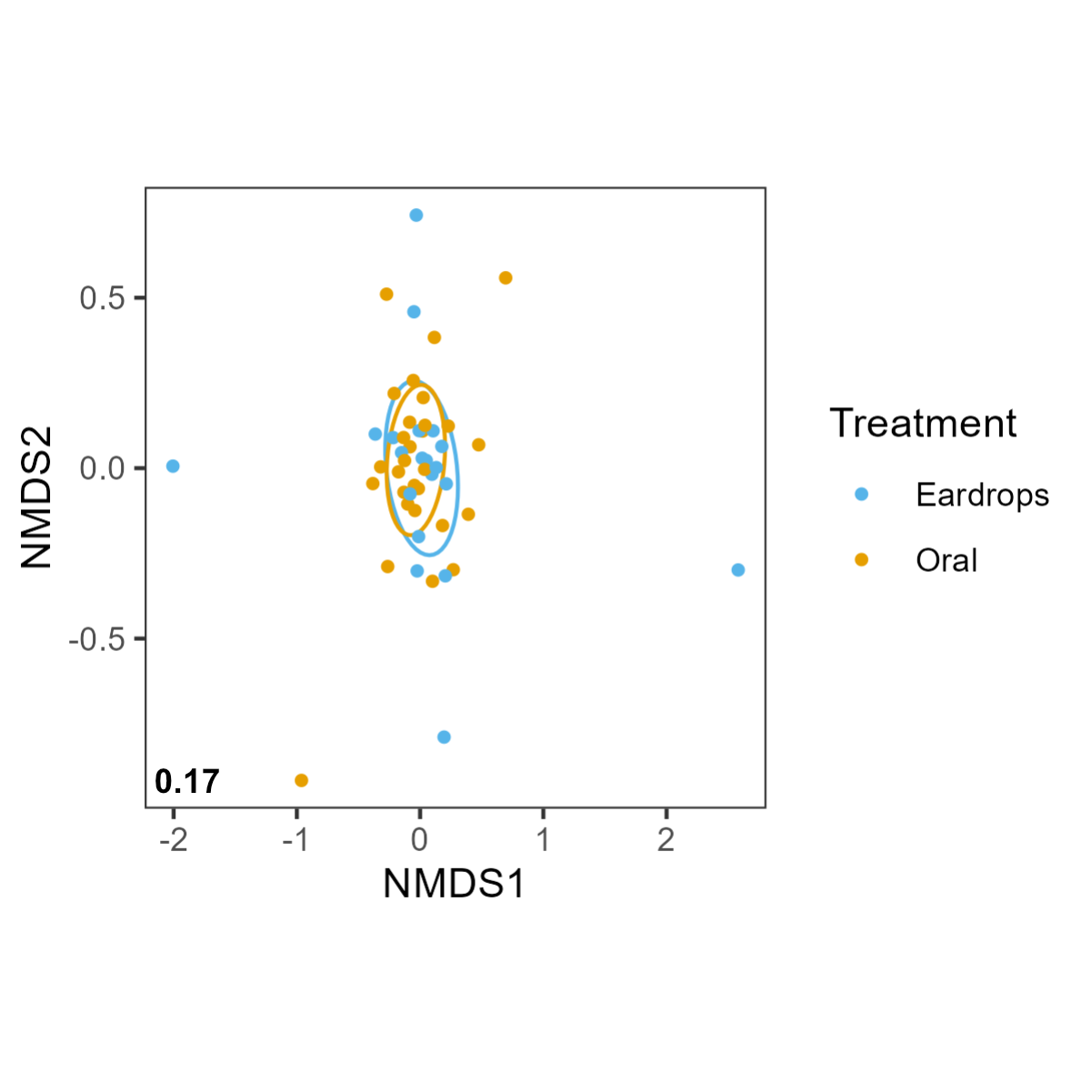


e) Total antibiotic resistance gene abundance within the gut microbiome^4^


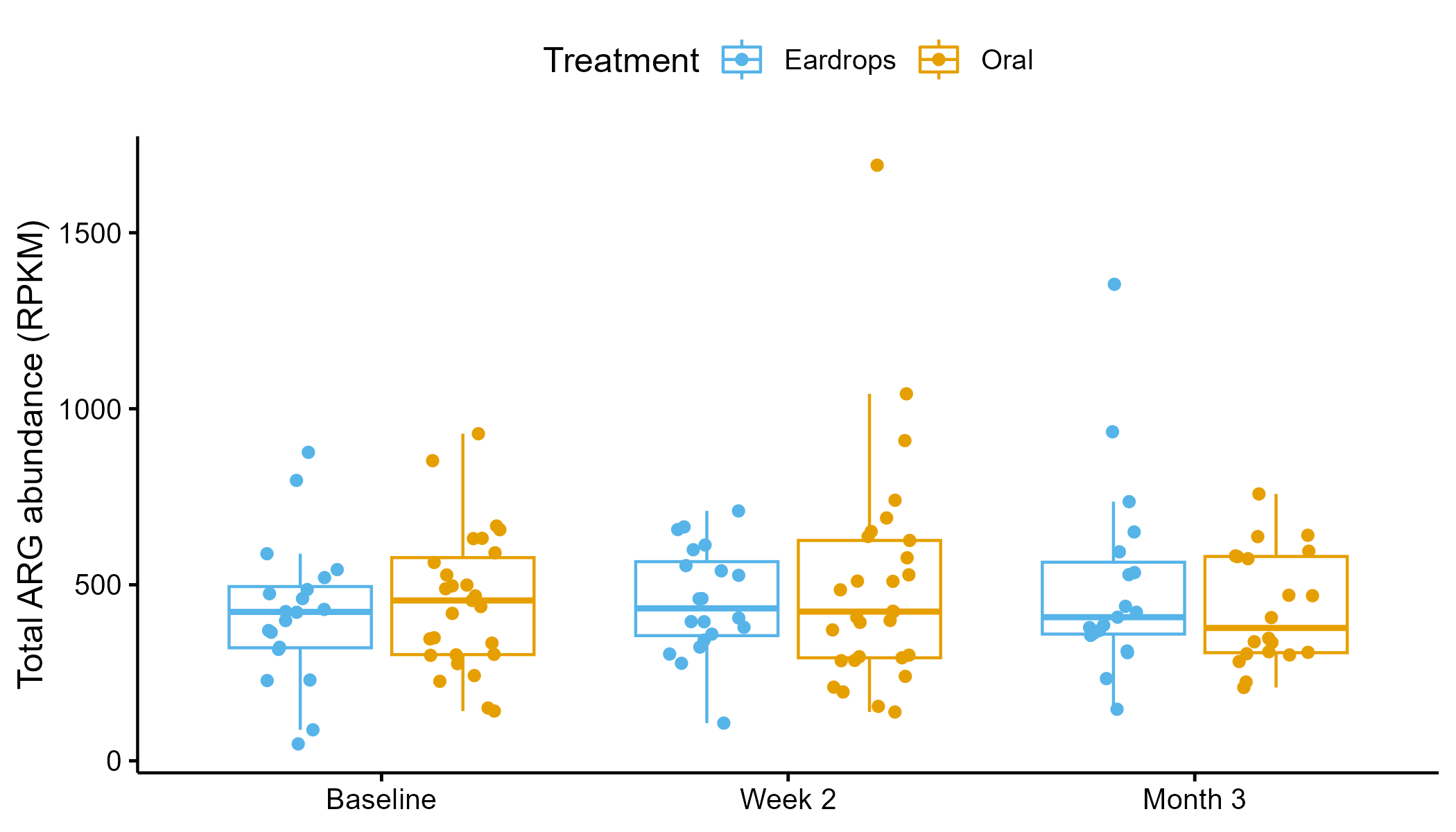


Abbreviations: AOMd= acute otitis media present with ear discharge due to spontaneous perforation of the eardrum; Eardrops=hydrocortisone-bacitracin-colistin eardrops; NMDS= non-metric multidimensional scaling; Oral=oral amoxicillin; Topical=hydrocortisone-bacitracin-colistin eardrops; RPKM= reads per kilobase of reference sequence per million sample reads.

1. The log10 + 1 transformed mean abundance RPKM of the 20 most abundant ARGs found in the gut microbiome samples. ARGs are clustered based on Euclidean distances. The RPKM values were summed across samples for each ARG. They were then ordered from most to least abundant and the top 20 most abundant genes were taken forward. The mean abundance of each timepoint for each treatment was log10 transformed and plotted.
2. Significance between treatment groups for alpha diversity was determined using the Wilcoxon rank sum test. Baseline p-value = 0.125 Week-2 p-value = 0.042, Month-3 p-value=0.667.
3. For beta-diversity, the 50% confidence interval ellipses are indicated. Stress is indicated within the plots. Significance was measured by PERMANOVA with 10,000 permutations.
4. Significance between treatment groups was determined using the Wilcoxon test. Baseline p-value = 0.572, Week-2 p-value = 0.864, Month-3 p-value=0.461.
