# Supplementary material for: The impact of topical or oral antibiotics in children with acute otitis media on their middle ear, nasopharyngeal, and gut microbiomes
Source: Epidemiol Infect. 2026 Jun 23;154:e94. doi: 10.1017/S0950268826101836 (PMC13366364; doi:10.1017/S0950268826101836)
Supplement: Claus et al. supplementary material [file S0950268826101836sup001.zip › 260225_Supplementary Table S1.docx]

**Table S1a: Baseline characteristics of all children that provided at least one sample during follow-up**

| **Characteristics, n (%)** | **All children sampled at least one during follow-up**  **N=57** | **Children that provided at least one MEF sample^1^**  **N=55** | **Children that provided at least one NP sample^2^**  **N=53** | **Children that provided at least one faecal sample^3^**  **N=50** |
| --- | --- | --- | --- | --- |
| **Age (mean (SD))** | 37.89 (27.14) | 36.60 (25.69) | 36.40 (26.92) | 33.48 (23.88) |
| **Sex, male** | 27 (47.4) | 27 (49.1) | 25 (47.2) | 24 (48.0) |
| **# previous AOMd episodes (mean (SD))^4^** | 1.72 (3.10) | 1.75 (3.16) | 1.83 (3.19) | 1.74 (3.15) |
| **>6 previous cold in past year^5^** | 31 (54.4) | 30 (54.5) | 28 (52.8) | 28 (56.0) |
| **Ever had ENT surgery** | 2 (3.5) | 2 (3.6) | 1 (1.9) | 1 (2.0) |
| **Diagnosed with asthma** | 8 (14.0) | 8 (14.5) | 7 (13.2) | 8 (16.0) |
| **Diagnosed with hay fever** | 1 (1.8) | 1 (1.8) | 1 (1.9) | 1 (2.0) |
| **Diagnosed with eczema** | 15 (26.3) | 15 (27.3) | 13 (24.5) | 13 (26.0) |
| **Smoker in household** | 4 (7.0) | 4 (7.3) | 4 (7.5) | 4 (8.0) |
| **Vaccinated with PCV** | 54 (94.7) | 52 (94.5) | 50 (94.3) | 49 (96.0) |
| **BL temperature (mean (SD))** | 37.59 (0.88) | 37.63 (0.91) | 37.57 (0.90) | 37.59 (0.89) |

Abbreviations: AOMd= acute otitis media present with ear discharge due to spontaneous perforation of the eardrum; BL temp= Temperature at baseline prior to treatment; ENT=ear nose throat; MEF=Middle ear fluid samples; NP=Nasopharyngeal samples; PCV= Pneumococcal conjugate vaccine; SD=standard deviation.

1. Of the 57 children that provided at least one MEF, NP, or faecal sample, 55 of them provided at least one MEF sample during follow-up, n=55 at baseline and n=5 at Week-2.
2. Of the 57 children that provided at least one MEF, NP, or faecal sample, 53 of them provided at least one NP sample during follow-up, n=53 at baseline and n=49 at Week-2.
3. Of the 57 children that provided at least one MEF, NP, or faecal sample, 50 of them provided at least one faecal sample during follow-up, n=47 at baseline and n=49 at Week-2 and n=39 at M3.
4. Number of episodes of ear discharge without ventilation tubes and excluding current episode.
5. Number of common colds in the previous 12 months, categorised as 0-6 colds versus more than 6 colds.

**Table S1b: Baseline characteristics of all children that provided at least one sample during follow-up by study treatment**

| **Characteristics, n (%)** | **MEF samples^1^**  **N=55** | | | **NP samples^2^**  **N=53** | | | **Faecal samples^3^**  **N=50** | | |
| --- | --- | --- | --- | --- | --- | --- | --- | --- | --- |
|  | **Ear**  **N=22** | **Oral**  **N=33** | **p** | **Ear**  **N=22** | **Oral**  **N=31** | **p** | **Ear**  **N=21** | **Oral**  **N=29** | **p** |
| **Age (mean (SD))** | 38.18 (26.71) | 35.55 (25.36) | 0.713 | 40.59 (30.91) | 33.42 (23.78) | 0.344 | 36.76 (28.73) | 31.10 (19.85) | 0.414 |
| **Sex, male** | 12 (54.5) | 15 (45.5) | 0.700 | 11 (50.0) | 14 (45.2) | 0.945 | 10 (47.6) | 14 (48.3) | 1.000 |
| **# previous AOMd episodes (mean (SD))^4^** | 1.64 (2.95) | 1.82 (3.33) | 0.837 | 1.73 (2.91) | 1.90 (3.42) | 0.845 | 1.71 (2.99) | 1.76 (3.32) | 0.961 |
| **>6 previous cold in past year^5^** | 11 (50.0) | 19 (57.6) | 0.782 | 10 (45.5) | 18 (58.1) | 0.531 | 11 (52.4) | 17 (58.6) | 0.881 |
| **Ever had ENT surgery** | 1 (4.5) | 1 (3.0) | 1.000 | 0 | 1 (3.2) | 1.000 | 0 | 1 (3.4) | 1.000 |
| **Diagnosed with asthma** | 4 (18.2) | 4 (12.1) | 0.815 | 3 (13.6) | 4 (12.9) | 1.000 | 4 (19.0) | 4 (13.8) | 0.913 |
| **Diagnosed with hay fever** | 1 (4.5) | 0 | 0.837 | 1 (4.5) | 0 | 0.862 | 1 (4.8) | 0 | 0.870 |
| **Diagnosed with eczema** | 5 (22.7) | 10 (30.3) | 0.757 | 5 (22.7) | 8 (25.8) | 1.000 | 4 (19.0) | 9 (31.0) | 0.513 |
| **Smoker in household** | 0 | 4 (12.1) | 0.244 | 0 | 4 (12.9) | 0.221 | 0 | 4 (13.8) | 0.213 |
| **Vaccinated with PCV** | 20 (90.9) | 32 (97.0) | 0.716 | 20 (90.9) | 30 (96.8) | 0.759 | 20 (95.2) | 28 (96.6) | 1.000 |
| **BL temperature (mean (SD))** | 37.54 (0.89) | 37.69 (0.94) | 0.548 | 37.43 (0.80) | 37.67 (0.97) | 0.346 | 37.51 (0.80) | 37.65 (0.96) | 0.603 |

Abbreviations: AOMd= acute otitis media present with ear discharge due to spontaneous perforation of the eardrum; BL temp= Temperature at baseline prior to treatment; Ear=hydrocortisone-bacitracin-colistin eardrops; ENT=ear nose throat; MEF=Middle ear fluid samples; NP=Nasopharyngeal samples; Oral=oral amoxicillin suspension; PCV= Pneumococcal conjugate vaccine; SD=standard deviation.

1. Of the 57 children that provided at least one MEF, NP, or faecal sample, 55 of them provided at least one MEF sample during follow-up, n=55 at baseline and n=5 at Week-2.
2. Of the 57 children that provided at least one MEF, NP, or faecal sample, 53 of them provided at least one NP sample during follow-up, n=53 at baseline and n=49 at Week-2.
3. Of the 57 children that provided at least one MEF, NP, or faecal sample, 50 of them provided at least one faecal sample during follow-up, n=47 at baseline and n=49 at Week-2 and n=39 at M3.
4. Number of episodes of ear discharge without ventilation tubes and excluding current episode.
5. Number of common colds in the previous 12 months, categorised as 0-6 colds versus more than 6 colds.
