# Supplementary material for: The impact of topical or oral antibiotics in children with acute otitis media on their middle ear, nasopharyngeal, and gut microbiomes
Source: Epidemiol Infect. 2026 Jun 23;154:e94. doi: 10.1017/S0950268826101836 (PMC13366364; doi:10.1017/S0950268826101836)
Supplement: Claus et al. supplementary material [file S0950268826101836sup001.zip › 260225_Supplementary_File_1.docx]

**The impact of topical or oral antibiotics in children with acute otitis media on their middle ear, nasopharyngeal, and gut microbiomes**

**Authors:**

Juana Claus, Ross S McInnes, Saskia Hullegie, Roger AMJ Damoiseaux, Anne GM Schilder, Janetta Top, Rob Schuurman, Mei Ling Chu, Debby Bogaert, Willem van Schaik, Roderick P Venekamp, Janneke HHM van de Wijgert

**Summary of PLOTS trial design and primary analysis**

The PLOTS trial was a primary care-based, open, individually randomized controlled non-inferiority trial comparing topical or oral antibiotics for children with acute otitis media (AOM) presenting with discharge (AOMd). The trial was registered in The Netherlands National Trial Register (NTR6723/NL6535; https://onderzoekmetmensen.nl/en/trial/23467) on 27 November 2017. Between December 2017 to February 2023, the trial was conducted in 52 primary care practices in the region Utrecht, the Netherlands, including 225 general practitioners (GPs). Recruitment was paused from 8 August 2018 to November 2021 due to supply issues with hydrocortisone-bacitracin-colistin eardrops and the COVID-19 pandemic. The trial was terminated early after enrolling 58 children due to slow recruitment. The trial’s rationale and design details have been described elsewhere[1] and primary analysis have been published following the CONSORT guidelines[2].

*Inclusion and exclusion criteria*

The following children were excluded: ventilation tubes in place or a pre-existing perforation of the eardrum, systemically very unwell, having received antibiotics during the previous two weeks and having had an episode of AOM in the previous 28 days, known immunodeficiency, craniofacial malformation, Down’s syndrome, previous ear surgery other than ventilation tubes, allergy to oral amoxicillin or hydrocortisone-bacitracin-colistin ear drops, and having already participated in the trial during a previous AOMd episode.

*Randomization*

An independent data manager generated a computer-generated randomization sequence with stratification according to age (<2 versus ≥2 years) and laterality (uni- versus bilateral AOM at baseline). After completing informed consent and baseline assessments, the trial doctor accessed a trial randomization website for concealed study group assignment. Assignment was balanced in a 1:1 ratio for the two study groups.

*Patient Involvement*

A parental panel was established. This parent panel was involved throughout all critical stages of the trial, including design and conduct of the research through regular parent panel meetings.

*Sample size calculation*

The clinically acceptable non-inferiority margin was set at 15%; that is, 50% of a 30% difference between oral antibiotics and placebo or no treatment as observed in earlier trials [3,4]. To demonstrate that the upper limit of a two-sided 95% confidence interval (CI) of the difference in treatment effect for the primary outcome does not exceed the predefined non-inferiority margin of 15% with 80% power, a minimum of 159 children per group was needed. To allow for 10% attrition, we aimed to randomize 350 children.

*Recruitment*

GPs informed parents of potentially eligible children about the trial, took consent to share their contact details with the study team at the UMC Utrecht and provided a study information leaflet. Upon receiving details, the trial doctor contacted parents by phone to provide detailed information about the study and scheduled a home visit on the same day for those who previously agreed to participate and whose child met the eligibility criteria.

*Treatment groups*

Children randomized to the oral antibiotic group were prescribed amoxicillin suspension, 50 mg per kilogram of body weight per day, divided over three doses administered orally for 7 days. Those randomized to eardrops were prescribed hydrocortisone-bacitracin colistin eardrops, five drops, three times per day in the discharging ear(s) for 7 days. Parents were instructed to clean the ear of any visible ear discharge with a tissue and apply the drops while tilting their child’s head to one side. During the three-month follow-up, any further treatment decisions were up to the GPs’ discretion.

*Statistical analysis of primary clinical outcomes*

The primary outcome was the proportion of children free from ear pain and fever on day 3 after randomization. All analyses were performed according to the intention-to-treat (ITT) principle. Further details have been published in main analysis paper [2].

In brief, children treated with eardrops (n = 26) had lower rates of symptom resolution (ear pain and fever) on day 3 than those receiving oral amoxicillin suspension (n = 31; 42% vs 65%; adjusted risk difference 20.3%, 95% CI −5.3% to 41.9%). Additionally, children in the eardrop group experienced longer parent-reported ear discharge (6 vs 3 days; p = .04) and slightly higher mean ear pain scores over days 1–3 (Likert scale 0–6: 2.1 vs 1.4; p = .02). However, children treated with eardrops required fewer oral antibiotic courses during the subsequent 3 months (11 courses in 25 children vs 33 courses in 30 children).

*Adverse events*

Discomfort during study medication administration was reported in 26% of children receiving eardrops compared to 19% receiving oral antibiotics. Gastro-intestinal upset occurred in 12% versus 32% of children in the eardrop and oral suspension groups, respectively. Body rashes occurred in 8% versus 16%, respectively. No serious adverse events were reported during the 3 months of follow-up.

**Supplementary methods**

*Processing and 16S rRNA sequencing of MEF and NP samples*

DNA extraction, 16S rRNA gene amplification, and sequencing were conducted following the protocol established by Odendaal et al.[1] In summary, DNA was isolated using the Mag Mini DNA Isolation Kit (ImmunoSource, Schilde, Belgium), with the use of mechanical and chemical cell lysis methods. The V4 hypervariable region of the 16S rRNA gene was amplified using primers 515F (5′-GTGCCAGCMGCCGCGGTAA-3′) and 806R (5′-GGACTACHVGGGTWTCTAAT-3′), which included Illumina adapter sequences and barcodes (Illumina Inc., San Diego, CA, USA). PCR amplification was performed with an initial denaturation at 98 °C for 30 seconds, followed by 30 cycles of 98 °C for 10 seconds, then 55 °C for 30 seconds, and 72 °C for 30 seconds, concluding with a final 5 minutes at 72 °C. Samples with DNA concentrations below 20 pg/μl were used undiluted while those with higher concentrations were diluted with HPLC-grade water (Instruchemie, Delfzijl, the Netherlands) accordingly. The pooled amplicons libraries were purified using AMPure XP reagent (Beckman Coulter Diagnostics, Brea, CA, USA) and quantified prior to sequencing. Sequencing was performed on the Illumina MiSeq platform using the MiSeq Reagent Kit v3 (2 × 300 bp paired-end reads; Illumina Inc., San Diego, CA, USA).

The raw sequencing data were processed using the DADA2 [2] R-package and annotated using the SILVA [3] v138.2 reference database. The resulting amplicon sequence variant (ASV) table was manually cleaned, and ASVs were agglomerated to genus level.

Mock community controls (ZymoBIOMICS Microbial Community Standard, D6300) and DNA controls (ZymoBIOMICS Microbial Community DNA Standard, D6305) from Zymo research (California, USA), as well as blanks from the DNA extraction and sequencing (Supplementary File 1), were included in the 16S rRNA amplicon sequencing procedures. All controls were processed in the same manner as the study samples and yielded the expected distribution profiles. Based on ASVs present in blank controls, we removed *Caulobacteraceae*, *Mesorhizobium*, *Sphingomonas*, *Acinetobacter*, and *Bradyrhizobium*.

We manually removed unassigned amplicon sequence variants (ASVs) and those assigned to mitochondria and chloroplast or broad taxonomic domains (Eukaryotes, Archaea, or Bacteria) from the raw ASV table. We used the National Center for Biotechnology Information Basic Local Alignment Search Tool (NCBI BLAST) for all ASVs with more than 500 reads that were not resolved at the genus level. All 16s rRNA controls were processed in the same manner as the study samples. Positive controls generated the expected sequencing results distributions. Contaminants (identified in the negative controls) were removed from the ASV tables prior to further analyses (see supplementary methods for more details). Singletons, defined as taxa that appears only once in one sample, were identified and removed.

*Testing of NP samples for respiratory tract viruses*

Multiplex realtime PCR assays (Allpex respiratory assays 1, 2 &3, Seegene, Seoul, South Korea) were used to test NP and MEF samples for the presence of common respiratory tract viruses: Adenovirus, Enterovirus, Human bocavirus, Human metapneumovirus, Influenza A (Pdmo9, H1, H3), Influenza B, Parainfluenza (1,2,3, and 4), Respiratory syncytial virus (A and B), Rhinovirus, Seasonal coronaviruses (OC43, 229E, and NL63). Additionally, NP and MEF samples were tested for SARS-CoV-2 with the Seegene Allplex 2019 nCoV assay (Seoul, South Korea). We combined all viruses (any virus present or no virus present) in the analyses due to the low prevalence of each individual virus.

*Processing and metagenomic sequencing of faecal samples*

For DNA isolation, an aliquot of ∼50 mg of faecal matter was defrosted, and DNA was isolated using the Agowa Mag DNA extraction kit (LGC genomics, Berlin, Germany). Libraries were prepared according to the DNA Nano library preparation protocol by Illumina, starting by shearing 100ng of gDNA with the Covaris S2 system using the 350bp settings stated in the Illumina protocol. After the library preparation libraries were checked with the Fragment Analyzer system dsDNA 910 Reagent Kit (35-1500bp) (Thermo Fisher Scientific, Waltmam, Massachusetts, USA) and with Qubit dsDNA HS Assay Kit (Qubit dsDNA HS Assay Kit). Libraries with a major peak of 350 bp and a DNA concentration of 4 ng/ul were taken forward for sequencing. Sample libraries were pooled in equimolar amounts and sequenced on a Novaseq 6000 (Illumina) with a S2 flowcell using the 150bp paired-end protocol.

Sequencing adapter removal and quality trimming was performed with fastp (v.0.23.2) using the default parameters.[4] The quality trimmed reads were then mapped against the human genome assembly (GRCh38: GCA_000001405.15) with bowtie 2 (v.2.4.1),[5] and SAMtools (v.1.5)[6] and BEDTools (v.2.26.0)[7] were used to retain the reads that did not map to the human genome assembly. No controls were used for the metagenomic sequencing procedures, as faecal samples are high biomass samples and undergo limited amplification during library preparation.

Community profiling was performed with MetaPhlAn4 (v.4.0.6) [8], using the ChocoPhlAn reference database (v.vOcCOVID2_CHOCOPhlAnSGB_202212). Reads that did not map to clade-specific markers were discarded. Profiling of antibiotic resistance genes (ARGs) was performed using ShortBRED [9]. Unique gene markers were identified using ShortBRED-Identify with the ResFinder antibiotic resistance gene database [10] and the reference database Uniref90 [11]. ShortBRED-Quantify was then used to map the metagenomic reads to the markers and obtain the RA of each gene, expressed as reads per kilobase of reference sequence per million sample reads (RPKM).

*Definition of responders versus non-responders*

Responders were defined as having lower *Streptococcus* EC at Week-2 than at baseline. We focused on *Streptococcus* because the study treatments target Gram-positive bacteria including streptococci, *S. pneumoniae* is considered an important AOM pathobiont [12], and the differential abundance analysis identified *Streptococcus* as the genus with the largest reductions after the treatments.

**Supplementary results**

*Results of respiratory tract virus panel testing*

Post-pandemic NP samples tested positive for at least one virus included in the viral panel less often (MEF baseline: 30.0%; NP baseline: 30.3%; NP Week-2: 51.5%) than pre-pandemic samples (MEF baseline: 70.0%; NP baseline: 69.7%; NP Week-2: 48.5%), although these differences were not statistically significant. Of the four children who still had MEF at Week-2, only one sample tested positive for at least one virus. In our study, the most common virus detected overall was rhinovirus.

*Impact of treatments on the gut microbiome composition and gut resistome*

A total of 117 ARGs were identified in the faecal samples. Tetracycline resistance genes were the most abundant class of ARGs, accounting for 45% (9/20) of the most abundant genes (Figure A.3). The most abundant ARG was tet(Q)_3. Other clinically relevant ARGs included the beta-lactamase gene blaTEM, and the aminoglycoside resistance genes ant(6)-Ia and aph(3_)-IIIa. Notably, four distinct erythromycin resistance genes were also among the most abundant.

*Responders vs. non-responders*

Baseline characteristics were compared between responders and non-responders, including prior AOMd episodes, frequency of colds in the previous year, history of ENT surgery, diagnoses of asthma, hay fever, and eczema, presence of a household smoker, and pneumococcal conjugate vaccine (PCV) status. Non-responders reported a higher number of previous AOMd episodes, although this was not statistically significant (2.44 in non-responders vs. 1.58 in responders; p=0.398).

**References**

1. Hasrat, R. *et al.* Benchmarking laboratory processes to characterise low-biomass respiratory microbiota. *Sci. Rep.* **11**, 17148 (2021).

2. Callahan, B. J. *et al.* DADA2: High-resolution sample inference from Illumina amplicon data. *Nat. Methods* **13**, 581–583 (2016).

3. Quast, C. *et al.* The SILVA ribosomal RNA gene database project: improved data processing and web-based tools. *Nucleic Acids Res.* **41**, D590–D596 (2013).

4. Chen, S., Zhou, Y., Chen, Y. & Gu, J. fastp: an ultra-fast all-in-one FASTQ preprocessor. *Bioinformatics* **34**, i884–i890 (2018).

5. Langmead, B. & Salzberg, S. L. Fast gapped-read alignment with Bowtie 2. *Nat. Methods* **9**, 357–359 (2012).

6. Li, H. *et al.* The Sequence Alignment/Map format and SAMtools. *Bioinformatics* **25**, 2078–2079 (2009).

7. Quinlan, A. R. & Hall, I. M. BEDTools: a flexible suite of utilities for comparing genomic features. *Bioinformatics* **26**, 841–842 (2010).

8. Blanco-Míguez, A. *et al.* Extending and improving metagenomic taxonomic profiling with uncharacterized species using MetaPhlAn 4. *Nat. Biotechnol.* **41**, 1633–1644 (2023).

9. Kaminski, J. *et al.* High-Specificity Targeted Functional Profiling in Microbial Communities with ShortBRED. *PLOS Comput. Biol.* **11**, e1004557 (2015).

10. Florensa, A. F., Kaas, R. S., Clausen, P. T. L. C., Aytan-Aktug, D. & Aarestrup, F. M. ResFinder – an open online resource for identification of antimicrobial resistance genes in next-generation sequencing data and prediction of phenotypes from genotypes. *Microb. Genomics* **8**, (2022).

11. Suzek, B. E., Huang, H., McGarvey, P., Mazumder, R. & Wu, C. H. UniRef: comprehensive and non-redundant UniProt reference clusters. *Bioinformatics* **23**, 1282–1288 (2007).

12. Horhat, R., Horhat, F.-R. & Mocanu, V. Is Multidrug Resistance in Acute Otitis Media with Streptococcus pneumoniae Associated with a More Severe Disease? *Med. Princ. Pract. Int. J. Kuwait Univ. Health Sci. Cent.* **30**, 571–578 (2021).
