# Supplementary material for: The impact of topical or oral antibiotics in children with acute otitis media on their middle ear, nasopharyngeal, and gut microbiomes
Source: Epidemiol Infect. 2026 Jun 23;154:e94. doi: 10.1017/S0950268826101836 (PMC13366364; doi:10.1017/S0950268826101836)
Supplement: Claus et al. supplementary material [file S0950268826101836sup001.zip › 260225_Supplementary_File_2.docx]

**Sensitivity analysis for NP samples**

Sensitivity analysis excluding participants who received additional antibiotics during follow-up.

**Figure B.1: Paired analyses of total bacterial load and log_10_ estimated concentrations of bacterial groups in nasopharyngeal samples before and after eardrop or oral suspension treatment in children with AOMd^1
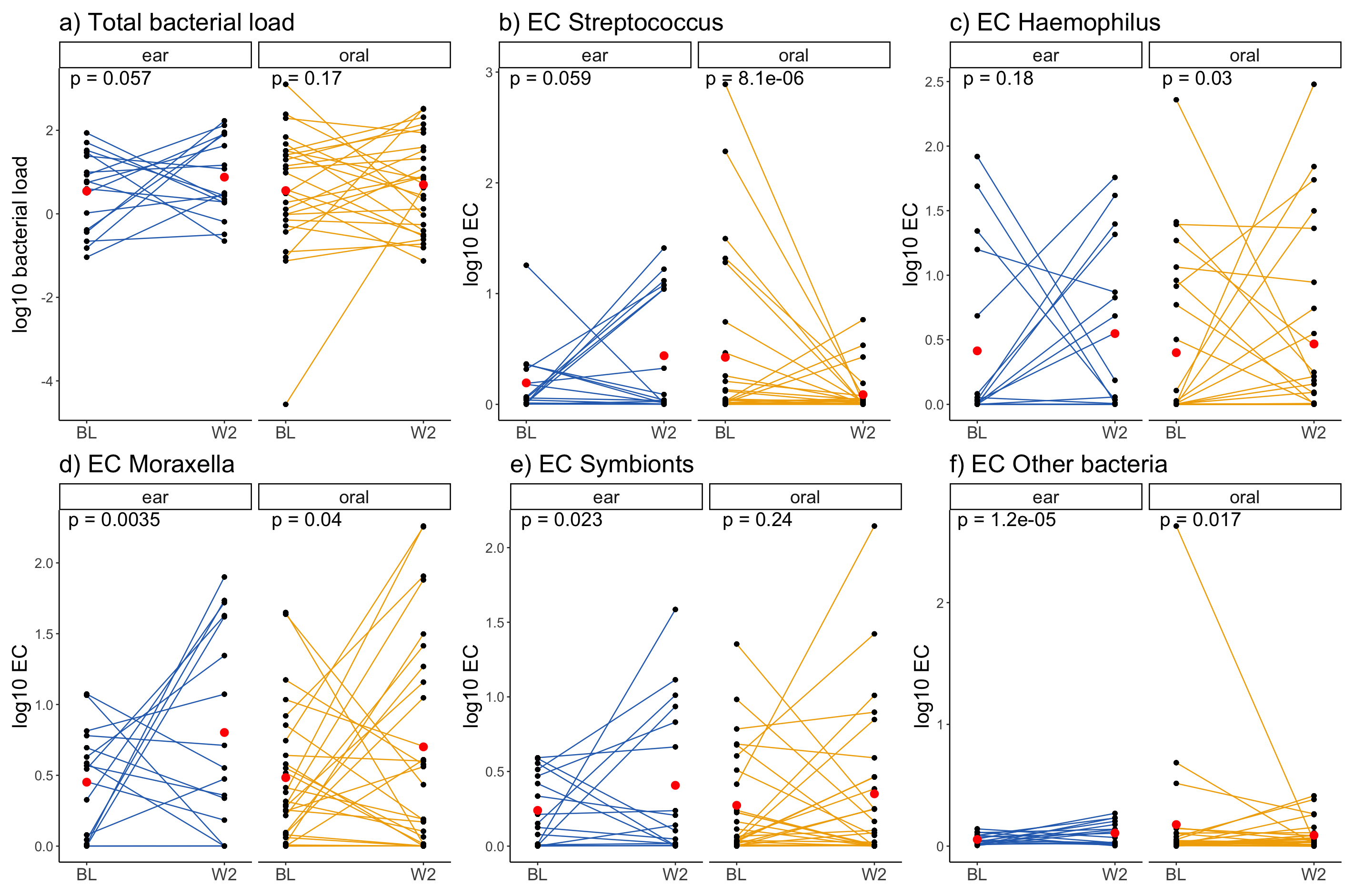
^**

Abbreviations: BL=baseline sampling timepoint; Ear=hydrocortisone-bacitracin-colistin eardrops; EC=log10 estimated concentration in pg/ul; Oral=oral amoxicillin; W2=week 2 sampling timepoint.

1. p-value method= **Wilcoxon rank-sum test (Mann-Whitney U test) for paired samples.** N=44 children with both baseline and Week-2 samples that did not receive additional antibiotics (n=17 ear and n=27 oral)

**Table B.1: Bivariable linear regression models with estimated concentration of one bacterial group in nasopharyngeal samples from children with AOMd^1^ as the outcome of each model**

| **Outcome** | **Determinant^2^** | **Coefficient (95% CI)** | **p-value** |
| --- | --- | --- | --- |
| Total bacterial load | W2 ear vs. baseline all | 0.28 (-0.38, 0.95) | 0.394 |
|  | W2 oral vs. baseline all | 0.10 (-0.46, 0.67) | 0.714 |
|  | W2 oral vs. W2 ear | -0.18 (-0.84, 0.48) | 0.682 |
| EC log10 Streptococcus | W2 ear vs. baseline all | 0.10 (-0.19, 0.39) | 0.505 |
|  | W2 oral vs. baseline all | -0.25 (-0.50, -0.01) | **0.045** |
|  | W2 oral vs. W2 ear | -0.35 (-0.58, -0.12) | **0.004** |
| EC log10 Haemophilus | W2 ear vs. baseline all | 0.11 (-0.27, 0.50) | 0.561 |
|  | W2 oral vs. baseline all | 0.03 (-0.30, 0.36) | 0.845 |
|  | W2 oral vs. W2 ear | -0.08 (-0.51, 0.34) | 0.704 |
| EC log10  Moraxella | W2 ear vs. baseline all | 0.30 (-0.04, 0.65) | 0.082 |
|  | W2 oral vs. baseline all | 0.20 (-0.09, 0.49) | 0.174 |
|  | W2 oral vs. W2 ear | -0.10 (-0.56, 0.36) | 0.658 |
| EC log10  Symbionts | W2 ear vs. baseline all | 0.15 (-0.09, 0.38) | 0.216 |
|  | W2 oral vs. baseline all | 0.09 (-0.11, 0.29) | 0.378 |
|  | W2 oral vs. W2 ear | -0.06 (-0.38, 0.26) | 0.722 |
| EC log10  Other | W2 ear vs. baseline all | -0.02 (-0.18, 0.14) | 0.812 |
|  | W2 oral vs. baseline all | -0.04 (-0.18, 0.10) | 0.588 |
|  | W2 oral vs. W2 ear | -0.02 (-0.08, 0.05) | 0.565 |

Abbreviations: CI= Confidence interval; Ear=hydrocortisone-bacitracin-colistin eardrops; EC=log10 estimated concentration in pg/ul; Oral=oral amoxicillin; W2=week 2 sampling timepoint.

1. At baseline, N=47 children (BL ear=18, BL oral=29). At Week-2, N=44 children (W2 ear=17, W2 oral=27).
2. Two regression models; 1) each treatment is compared to all baseline samples; 2) the Week-2 samples are compared to each other; eardrops vs. oral suspension.

**Sensitivity analysis for faecal samples**

**Figure B.3: The 20 most abundant antibiotic resistance genes present in the gut microbiome samples of children undergoing treatment for acute otitis media**


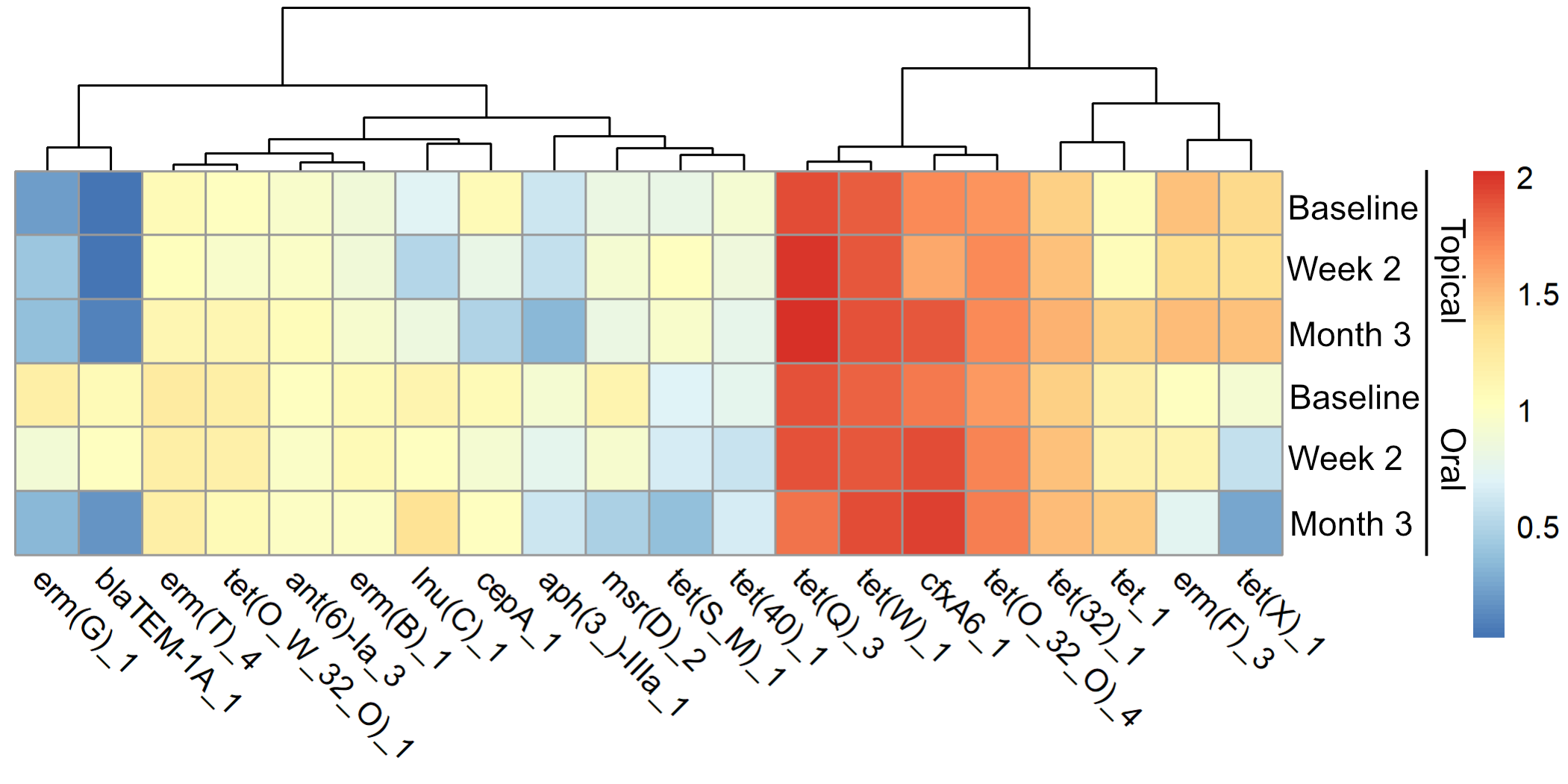


Abbreviations: AOMd= acute otitis media present with ear discharge due to spontaneous perforation of the eardrum; Topical=hydrocortisone-bacitracin-colistin eardrops; Oral=oral amoxicillin; RPKM= reads per kilobase of reference sequence per million sample reads.

The log10 + 1 transformed mean abundance RPKM of the 20 most abundant ARGs found in the gut microbiome samples. ARGs are clustered based on Euclidean distances.

**Figure B.4: Alpha-diversity of antibiotic resistance genes present within** **the gut microbiome of children undergoing treatment for acute otitis media**

*
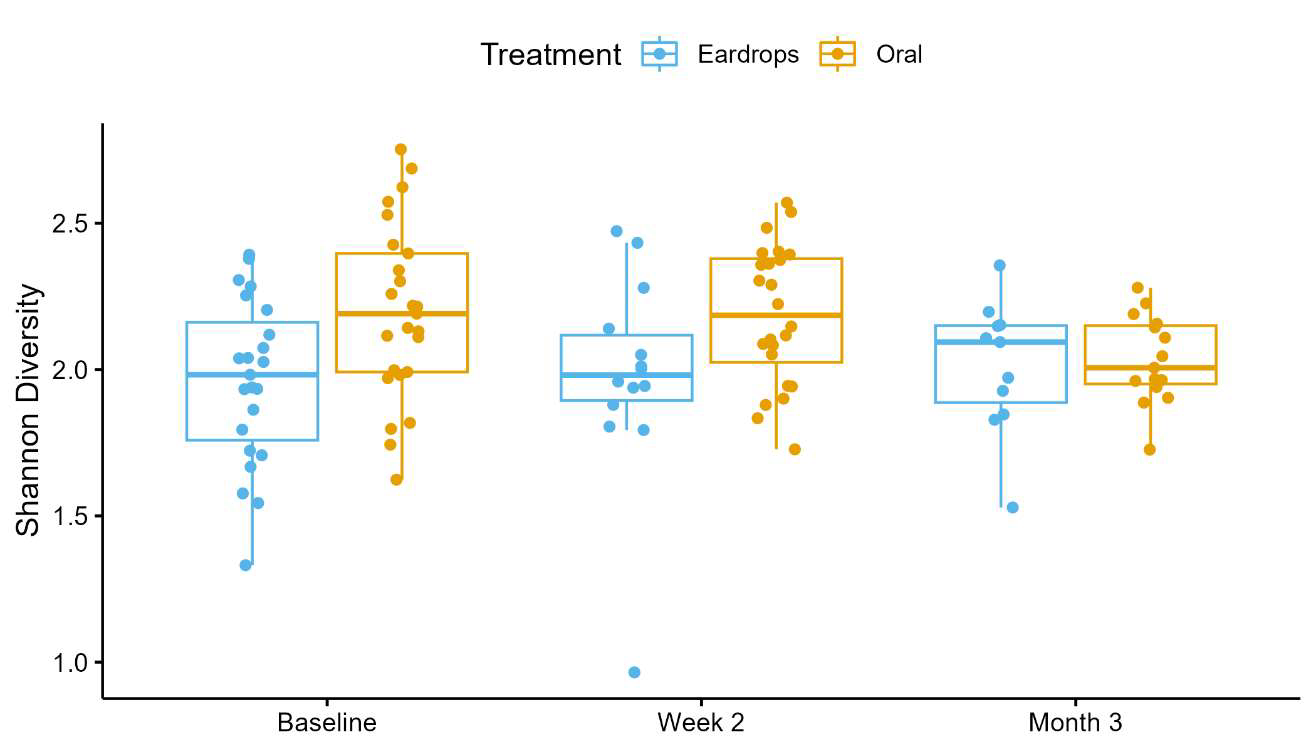
*

The alpha diversity was measured using the Shannon diversity index. Significance was determined using the Wilcoxon test. Baseline p-value = 0.012, Week-2 p-value = 0.054, Month-3 p-value=0.919.

**Figure B.5: Beta-diversity of antibiotic resistance genes present within the gut microbiome of children undergoing treatment for acute otitis media**

**a) Samples prior to treatment b) Samples one week post treatment**

*
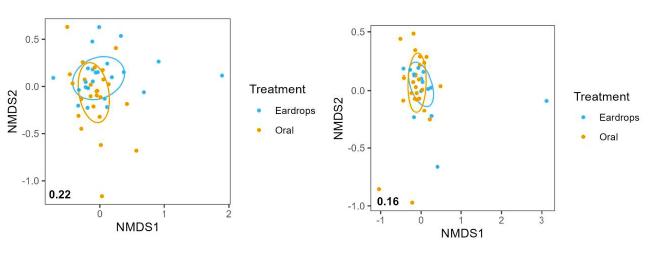
*

Non-metric multidimensional scaling was performed on Bray-Curtis dissimilarity matrices generated from antibiotic resistance gene abundances. 50% confidence interval ellipses are indicated. Stress is indicated within the plots. Significance was measured by PERMANOVA with 10,000 permutations.

**Figure B.6: Total antibiotic resistance gene abundance within the gut** **microbiome of children undergoing treatment for acute otitis media**

*
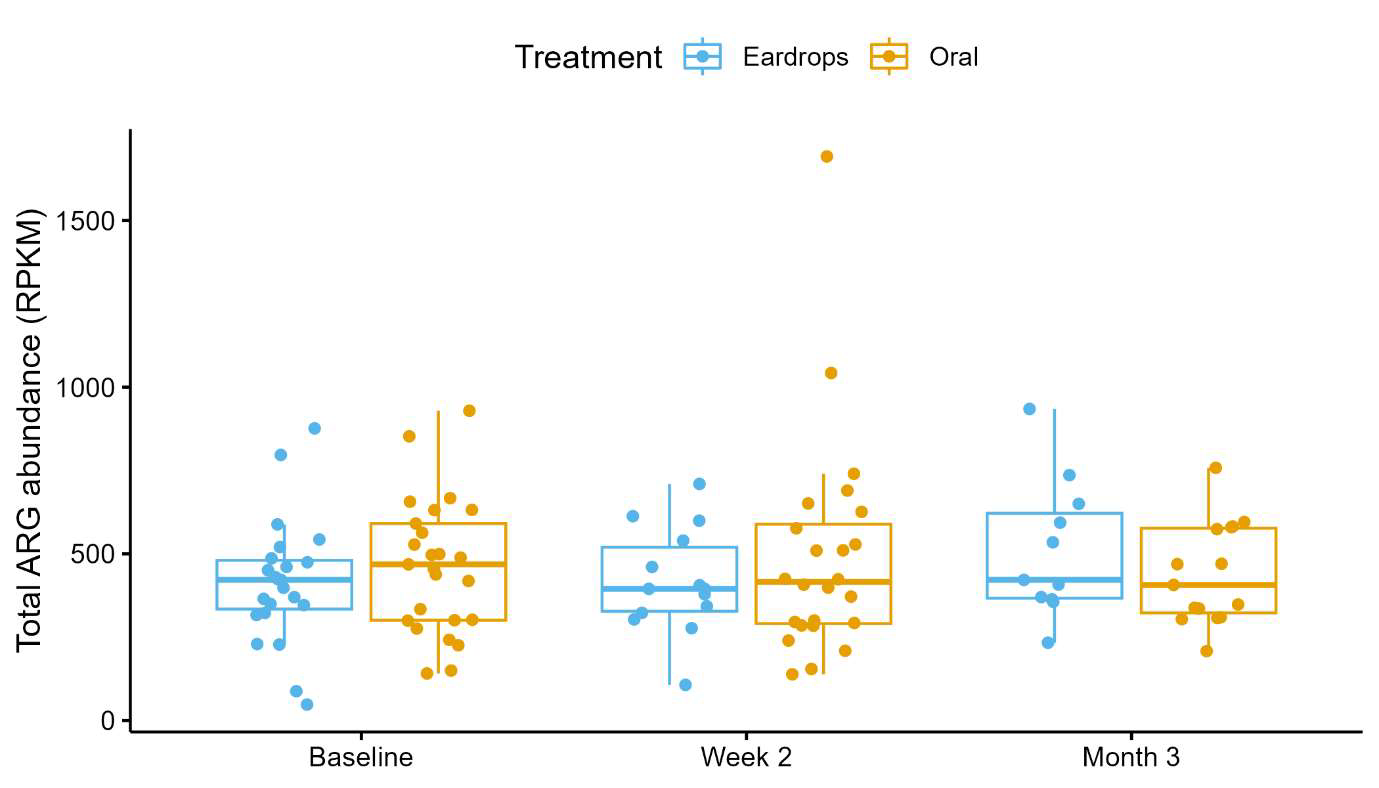
*

Abbreviations: AOMd= acute otitis media present with ear discharge due to spontaneous perforation of the eardrum; Ear=hydrocortisone-bacitracin-colistin eardrops; Oral=oral amoxicillin; RPKM= reads per kilobase of reference sequence per million sample reads

Significance was determined using the Wilcoxon test. Baseline p-value = 0.378, Week-2 p-value = 0.823, Month-3 p-value=0.281.
